# Supplementary material for: The causes and consequences of Alzheimer’s disease: phenome-wide evidence from Mendelian randomization
Source: Nat Commun. 2022 Aug 11;13:4726. doi: 10.1038/s41467-022-32183-6 (PMC9372151; doi:10.1038/s41467-022-32183-6)
Supplement: Supplementary file 1 — Supplementary Information [file 41467_2022_32183_MOESM1_ESM.pdf]

Supplementary Information

The causes and consequences of Alzheimer's disease: phenome-wide evidence from Mendelian randomization

Roxanna Korologou-Linden, MSc[1,2], Laxmi Bhatta, PhD[3], Ben Brumpton, PhD[3,4, 5], Laura D Howe, PhD[1,2], Louise A C Millard, PhD[1,2,6], Katarina Kolaric, PhD[1,2], Yoav Ben-Shlomo, PhD[2], Dylan M Williams, PhD[7,8], George Davey Smith, FRS[1,2], Emma L Anderson[†], PhD[1,2], Evie Stergiakouli[†], PhD[1,2], Neil M Davies[†], PhD[1,2,3]

[1] Medical Research Council Integrative Epidemiology Unit, Bristol Medical School, University of Bristol, BS8 2BN, United Kingdom.

[2] Population Health Sciences, Bristol Medical School, University of Bristol, Barley House, Oakfield Grove, Bristol, BS8 2BN, United Kingdom.

[3] K.G. Jebsen Center for Genetic Epidemiology, Department of Public Health and Nursing, NTNU, Norwegian University of Science and Technology, Trondheim, Norway.

[4] Clinic of Medicine, St. Olavs Hospital, Trondheim University Hospital, Trondheim, Norway.

[5] HUNT Research Center, Department of Public Health and Nursing, NTNU, Norwegian University of Science and Technology, Levanger, Norway.

[6] Intelligent Systems Laboratory, Department of Computer Science, University of Bristol, Bristol, UK

[7] MRC Unit for Lifelong Health and Ageing at UCL, University College London, London, UK.

[8] Department of Medical Epidemiology & Biostatistics, Karolinska Institutet, Stockholm, Sweden.

Supplementary Table of Contents

Supplementary Methods 1

Alzheimer's disease GWAS 1
UK BIOBANK 1
IGAP 1
Psychiatric Genetics Consortium 2
Alzheimer's Disease Sequencing Project 2
Meta-analysis of samples 3

Target sample for polygenic risk score 3
Supplementary Figure 1. UK Biobank participant flow diagram 4
Polygenic risk score 4
Supplementary Figure 2. Flow diagram showing SNP selection used to generate the polygenic risk score 5
Supplementary Table 1: SNPs reaching genome-wide significance in meta-analysis of IGAP, PGC, and ADSP 6

PHESANT 7
Supplementary Table 2: UK Biobank fields excluded from PheWAS 7
Supplementary Table 3: Description of ordered categorical variables in PHEWAS and MR analyses 8

Risk factors implicated in Alzheimer's disease in previous research 11

Replication 11

Follow-up using MR 14
Supplementary Table 4: Sample size of cases and controls for binary phenotypes used in Mendelian randomization analysis 15

Supplementary Note 1: Phenome-wide association study 17
Supplementary Figure 3. Forest plots showing effect estimates for the association between polygenic risk score including the *APOE* region, general medical history and dementia-associated medical history by age tertile 17
Supplementary Figure 4. Forest plots showing effect estimates for the association between polygenic risk score including the *APOE* region, family history and dietary choices by age tertile 18
Supplementary Figure 5. Forest plot showing the effect estimates for the association between the polygenic score for Alzheimer's disease (including the *APOE* region), lifestyle, and previously implicated risk factors for Alzheimer's disease 19
Supplementary Figure 6. Forest plot showing the age-stratified effect estimates for the association between the polygenic score for Alzheimer's disease (including the APOE region), and participant medical history in UK Biobank (diamond markers) and HUNT (replication sample, circle markers). 21
Supplementary Figure 7. Forest plot showing the age-stratified effect estimates for the association between the polygenic score for Alzheimer's disease (including the APOE region) and family medical history in UK Biobank (diamond markers) and HUNT (replication sample, circle markers). 22
Supplementary Figure 8. Forest plot showing the age-stratified effect estimates for the association between the polygenic score for Alzheimer's disease (including the APOE region) and dietary habits in UK Biobank (diamond markers) and HUNT (replication sample, circle markers). 23

Supplementary Figure 9. Forest plot showing the age-stratified effect estimates for the association between the polygenic score for Alzheimer's disease (including the *APOE* region) and dietary habits in UK Biobank (diamond markers) and HUNT (replication sample, circle markers) 24
Supplementary Figure 10. Forest plot showing the age-stratified effect estimates for the association between the polygenic score for Alzheimer's disease (including the *APOE* region) and lifestyle in UK Biobank (diamond markers) and HUNT (replication sample, circle markers) 25
Supplementary Figure 11. Forest plots showing effect estimates for the association between polygenic risk score including SNPs in the *APOE* region, medical history and dementia-associated medical history in the entire UK Biobank sample. 26
Supplementary Figure 12. Forest plots showing effect estimates for the association between polygenic risk score including SNPs in the *APOE* region, parental health factors, and physical measures in the entire UK Biobank sample. 28
Supplementary Figure 13. Forest plots showing effect estimates for the association between polygenic risk score including SNPs in the *APOE* region, biological measures, brain-related and cognitive test measures in the entire UK Biobank sample. 29
Supplementary Figure 14. Forest plots showing effect estimates for the association between polygenic risk score including SNPs in the *APOE* region, dietary choices, and lifestyle in the entire UK Biobank sample. 30
Supplementary Figure 15. Forest plots showing effect estimates for the association between polygenic risk score excluding *APOE* (for top hits when SNPs in the *APOE* region were included), medical history, and dementia-associated medical history in the entire UK Biobank sample 32
Supplementary Figure 16. Forest plots showing effect estimates for the association between polygenic risk score excluding *APOE* (for top hits when SNPs in the *APOE* region were included), parental health factors, and physical measures in the entire UK Biobank sample 34
Supplementary Figure 17. Forest plots showing effect estimates for the association between polygenic risk score excluding *APOE* (for top hits when SNPs in the *APOE* region were included), biological, brain-related and cognitive test measures in the entire UK Biobank sample 35
Supplementary Figure 18. Forest plots showing effect estimates for the association between polygenic risk score excluding *APOE* (for top hits when SNPs in the *APOE* region were included), dietary choices, and lifestyle measures in the entire UK Biobank sample 36
Supplementary Table 5: Detailed description of multinomial outcome estimates for the PheWAS using the polygenic risk score including the APOE region 37
Supplementary Table 6: Detailed description of multinomial outcome estimates for the PheWAS using the polygenic risk score excluding the *APOE* region 38
Supplementary Table 7: Detailed description of multinomial outcome estimates for the PheWAS using the polygenic risk score including the *APOE* region in tertile 1 39
Supplementary Table 8: Detailed description of multinomial outcome estimates for the PheWAS using the polygenic risk score including the *APOE* region in tertile 2 40
Supplementary Table 9: Detailed description of multinomial outcome estimates for the PheWAS using the polygenic risk score including the *APOE* region in tertile 3 41

Supplementary Note 2: Follow-up using Mendelian randomization 42
Supplementary Table 10: Effect estimates with 95% confidence intervals examining the causal association between family medical history and Alzheimer's disease using Mendelian Randomization 43

Supplementary Table 11: Effect estimates with 95% confidence intervals examining the causal association between medical history and Alzheimer's disease using Mendelian Randomization 44
Supplementary Table 12: Effect estimates with 95% confidence intervals examining the causal association between physical measures and Alzheimer's disease using Mendelian Randomization 46
Supplementary Table 13: Effect estimates with 95% confidence intervals examining the causal association between cognitive and brain-related measures and Alzheimer's disease using Mendelian Randomization 48
Supplementary Table 14: Effect estimates with 95% confidence intervals examining the causal association between biological measures and Alzheimer's disease using Mendelian Randomization 49
Supplementary Table 15: Effect estimates with 95% confidence intervals examining the causal association between dietary choices and Alzheimer's disease using Mendelian Randomization 50
Supplementary Table 16: Effect estimates with 95% confidence intervals examining the causal association between lifestyle factors and Alzheimer's disease using Mendelian Randomization 52
Supplementary Table 17: Effect estimates with 95% confidence intervals examining the causal association between factors implicated in Alzheimer's disease in previous studies and Alzheimer's disease using Mendelian Randomization 53
Supplementary Figure 1. Plot displaying the effect of each corresponding SNP on basal metabolic rate (N=454,874) and Alzheimer's disease (N_{cases}=........ 55
Supplementary Figure 19. Plot displaying the effect of each corresponding SNP on forced vital capacity 56
Supplementary Figure 20. Plot displaying the effect of each corresponding SNP on whole body fat-free mass 57
Supplementary Figure 21. Plot displaying the effect of each corresponding SNP on whole body water mass 58
Supplementary Figure 22. Plot displaying the effect of each corresponding SNP on moderate physical activity (>10 minutes) 59
Supplementary Figure 23. Plot displaying the effect of each corresponding SNP on A-level qualifications 60
Supplementary Figure 24. Plot displaying the effect of each corresponding SNP on having a college degree 61
Supplementary references 62

Supplementary Methods

Alzheimer's disease GWAS

UK BIOBANK

The full data release contains the cohort of successfully genotyped samples (n=488,377). 49,979 individuals were genotyped using the UK BiLEVE array and 438,398 using the UK Biobank axiom array. Pre-imputation QC, phasing and imputation are described elsewhere [1]. In brief, prior to phasing, multiallelic SNPs or those with MAF ≤1% were removed. Phasing of genotype data was performed using a modified version of the SHAPEIT2 algorithm [2] . Genotype imputation to a reference set combining the UK10K haplotype and HRC reference panels[3] was performed using IMPUTE2 algorithms[4]. The analyses presented here were restricted to autosomal variants within the HRC site list using a graded filtering with varying imputation quality for different allele frequency ranges. Therefore, rarer genetic variants are required to have a higher imputation INFO score (Info>0.3 for MAF >3%; Info>0.6 for MAF 1-3%; Info>0.8 for MAF 0.5-1%; Info>0.9 for MAF 0.1-0.5%) with MAF and Info scores having been recalculated on an in-house derived 'European' subset [5].

IGAP

The International Genomics of Alzheimer's Project (IGAP) is a large two-stage study based upon genome-wide association studies (GWAS) on individuals of European ancestry. In stage 1, IGAP used genotyped and imputed data on 7,055,881 single nucleotide polymorphisms (SNPs) to meta-analyse four previously published GWAS datasets consisting of 17,008 Alzheimer's disease cases and 37,154 controls performed by GERAD (Genetic and Environmental Risk in Alzheimer's disease), EADI (The European Alzheimer's disease Initiative), CHARGE (Cohorts for Heart and Aging Research in Genomic Epidemiology), and ADGC (Alzheimer Disease Genetics Consortium) [6]. Complete details of each study, as well as the samples and methodologies are reported elsewhere [6–10]. Each

dataset was imputed with either Impute 2 [4] or MACH software [11], utilising the 1000 genomes data as a reference panel.

Psychiatric Genetics Consortium

Three non-public datasets (the Norwegian DemGene network, the Swedish Twin Studies of Aging and TwinGene) were meta-analysed by the Alzheimer group initiative. Genetic data were collected from the Norwegian DemGene Network consisting of 2,224 cases and 1,855 healthy controls. The DemGene Study is a Norwegian network of clinical sites collecting cases from Memory Clinics on the basis of standardised examination of cognitive, functional and behavioural measures and data on progression of the majority of patients. A total 2,224 cases were diagnosed with AD from 7 studies: the Norwegian Register of persons with Cognitive Symptoms (NorCog), the Progression of Alzheimer's Disease and Resource use (PADR), the Dementia Study of Western Norway (DemVest), the AHUS study, the Dementia Study in Rural Northern Norway (NordNorge), the HUNT Dementia Study, the Nursing Home study, and the TrønderBrain study. These cases were diagnosed according to the recommendations from the National Institute on Aging–Alzheimer's Association (NIA/AA) (AHUS), the NINCDS-ADRDA criteria (DemVest and TrønderBrain) or the ICD-10 research criteria (NorCog, PADR, NordNorge and HUNT). The controls from Norway were obtained through the AHUS, NordNorge, HUNT and TrønderBrain studies. The controls were screened with standardized interview and cognitive tests. Genotypes of the 4079 individuals from the DemGene Study were obtained with Human Omni Express-24 v.1.1 (Illumina Inc., San Diego, CA, USA) at deCODE Genetics (Reykjavik, Iceland).

Alzheimer's Disease Sequencing Project

The Alzheimer's Disease Sequencing Project (ADSP) collaboration aims to identify that contribute to AD risk by studying genetic sequencing data. ADSP sequencing data is available through the Genotypes and Phenotypes database (dbGaP) under the study accession: phs000572.v7.p (https://www.ncbi.nlm.nih.gov/projects/gap/cgi779

bin/study.cgi?study_id=phs000572.v1 .p1). Access was obtained to 10,907 individuals (5,771 cases, 5,136 controls) with whole-exome sequencing data. A considerable number of participants of the ADSP cohort were previously also included in IGAP. To avoid inflated meta-analysis results due to sample overlap, ADSP individuals that were duplicates based on the comparison of individual level genetic data between IGAP and ADSP were excluded [12].

Meta-analysis of samples

The meta-analysis of the summary statistics of IGAP, PGZ-ALZ, and ADSP was conducted using the –meta-analysis + qt report-all command in PLINK 1.9, using the classical inverse variance approach which weights effect estimates by the sampling distribution. All SNPs of the meta-analysis output (including SNPs with the effect estimates derived from only one study) were used requiring that they had data on SNP identifier, chromosome position, A1, A2, the effect allele frequency, a standard error or a p-value.

Target sample for polygenic risk score

UK Biobank is a population-based study of 503,325 people recruited between 2006 and 2010 from across Great Britain [13,14].This work was done under application number 16729 (version 2 genetic data [500K with HRC imputation] and phenotype dataset 21753). In **Supplementary Fig 1**, the flowchart shows the number of participants removed at each stage of the quality control pipeline.

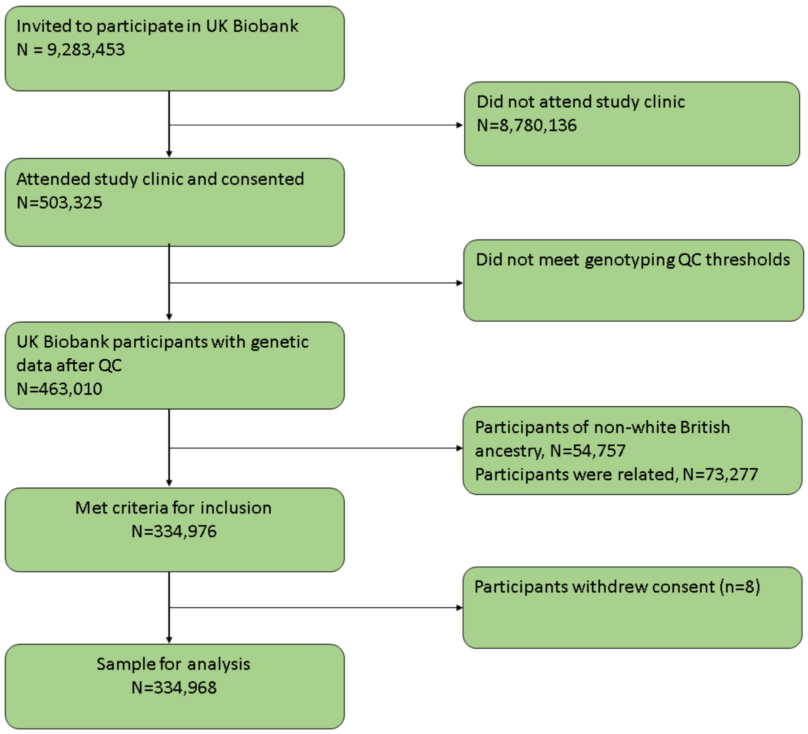

Supplementary Figure 1. UK Biobank participant flow diagram

Polygenic risk score

We constructed a standardised weighted PRS including single nucleotide polymorphisms (SNPs) associated with Alzheimer's disease at $p \leq 5 \times 10^{-8}$ for UK Biobank participants, based on the summary statistics from a meta-analysis of the IGAP consortium [6], ADSP [15] and PGC [16], totalling 24,087 individuals with a clinical diagnosis of Alzheimer's disease, paired with 55,058 controls. SNPs were clumped using r^2>0.001 and a physical distance for clumping of 10,000 kb. A polygenic risk score was calculated for each participant with genetic data using PLINK (version 1.9)[17]. Each score was calculated from the effect size (logarithm (log) odds)-weighted sum of associated alleles within each participant.

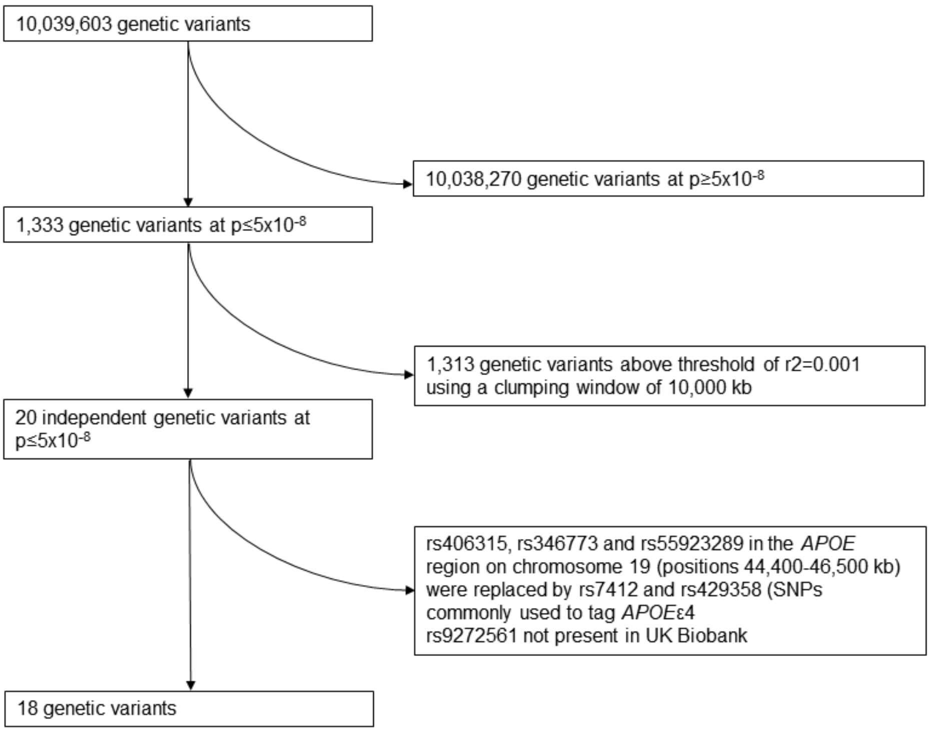

Supplementary Figure 2. Flow diagram showing SNP selection used to generate the polygenic risk score

Supplementary Table 1: SNPs reaching genome-wide significance in meta-analysis of IGAP, PGC, and ADSP

SNP	CHR	POS	A1*	A2	BETA	SE	P
rs2093760	1	207786828	A	G	0.148	0.016	$1.14x10^{-9}$
rs6733839	2	127892810	T	C	0.185	0.016	$2.60x10^{-30}$
rs7657553	4	11723235	A	G	0.088	0.016	$2.92x10^{-8}$
rs9272561	6	32607141	G	A	0.137	0.022	$8.75x10^{-10}$
rs9381563	6	47432637	C	T	0.092	0.015	$2.16x10^{-9}$
rs11763230	7	143108841	C	T	0.127	0.019	$7.25x10^{-12}$
rs1859788	7	99971834	G	A	0.094	0.015	$1.25x10^{-10}$
rs11787077	8	27465312	C	T	0.140	0.014	$2.09x10^{-23}$
rs11257242	10	11721119	G	C	0.087	0.016	$3.52x10^{-8}$
rs10792832	11	85867875	G	A	0.129	0.015	$5.90x10^{-18}$
rs11218343	11	121435587	T	C	0.2577	0.038	$1.51x10^{-11}$
rs7935829	11	59942815	A	G	0.110	0.013	$3.55x10^{-16}$
rs12590654	14	92938855	G	A	0.093	0.016	$1.09x10^{-8}$
rs2632516	17	56409089	G	C	0.087	0.015	$3.12x10^{-9}$
rs8093731	18	29088958	C	T	0.6136	0.112	$4.66x10^{-8}$
rs4147929	19	1063443	A	G	0.1219	0.021	$2.96x10^{-9}$
rs7412	19	45412079	C	T	0.4254	0.036	$2.63x10^{-32}$
rs429358	19	45411941	C	T	1.26	0.172	$2.53x10^{-13}$

*A1 refers to effect allele

†SNP was not included in polygenic risk score as not available in UK Biobank

PHESANT

The decision rule begins with the variable field type and use rules to categorise each variable as one of the four data types: continuous, ordered, categorical, unordered categorical or binary. Variables with the continuous and integer field type are typically assigned to the continuous data type, but some are assigned to ordered categorical, if, for example, there are only a few distinct values. Variables of the categorical (Single) field type are assigned to either the binary, ordered categorical or unordered categorical, depending on whether the field has two distinct values, or has been specified as ordered or unordered in the PHESANT setup files. Variables of the categorical (multiple) field type are converted to a set of binary variables, one for each of value in the categorical (multiple) fields) [18].

Supplementary Table 2: UK Biobank fields excluded from PheWAS

Number of fields	Field IDs	Reason for exclusion from phenome scan
1	54	Assessment centre
17	22000, 22001, 22003, 22004, 22005, 22006, 22009, 22010, 22011, 22012, 22013, 22018, 22019, 22021, 22027, 22051, 22052	Genetic data description fields
1	31	Sex field
5	34, 52, 21003, 21022, 21200	Age fields
17	20012, 20013, 20014, 3059, 3065, 3081, 4268, 4275, 4281, 4287, 5149, 5152, 5155, 5164, 6024, 6074, 6075	Assessment centre environment (ACE) fields
18	4232, 4243, 4259, 5090, 5091, 5136, 5138, 5139, 5140, 5141, 5142, 5143, 5144, 5145, 5146, 5147, 5148, 10691	Categorical (single) field with more than one value recorded per person.

Supplementary Table 3: Description of ordered categorical variables in PHEWAS and MR analyses

Alcohol intake versus 10 years previously	Self-reported alcohol intake versus 10 years previously<br>Ordered categorical variable (*More nowadays, about the same, less nowadays*)	1628
Cereal intake	Self-reported average weekly cereal intake (bowls)<br>Ordered categorical variable (n categories=3)	1458
Fluid intelligence score	Simple unweighted sum of the number of correct answers given to 13 fluid intelligence questions.<br>Ordered categorical variable (n categories=13)	20016
Fresh fruit intake	Self-reported average daily intake of fresh fruits (pieces) over the last year (counting one apple, one banana, 10 grapes, etc as one piece)<br>Ordered categorical variable (n categories=3)	1309
Frequency of walking for pleasure	Self-reported frequency of walking for pleasure in last 4 weeks<br>Ordered categorical variable (*Once in the last 4 weeks, 2-3 times in the last 4 weeks, once a week, 2-3 times a week, 4-5 times a week, everyday*).	971
Getting up in the morning	Self-reported ease of getting up in the morning in relation (if this varies a lot, in relation to the last 4 weeks)<br>Ordered categorical variable (*Not at all easy, not very easy, fairly easy, very easy).*	1170
Intake of sugar added to coffee	Self-reported intake of sugar added to tea (teaspoons)<br>Ordered categorical variable (n of categories=5)	100490
Intake of sugar added to coffee	Self-reported intake of sugar added to coffee (teaspoons)<br>Ordered categorical variable (n of categories=5)	100380
Lamb/mutton intake	Self-reported average intake of lamb/mutton intake (not including processed meats) considering intake over the last year.<br>Ordered categorical variable (*Never, less than once a week, once a week, 2-4 times a week, 5-6 times a week, once or more daily*)	1379
Nap during the day	Self-reported napping during the day<br>Ordered categorical variable (Never/rarely, sometimes, usually)	1190

Non-oily fish intake	Self-reported average non-oily fish intake (e.g. cod, tinned tuna, haddock) considering intake over the last year.<br>Ordered categorical variable (*Never, less than once a week, once a week, 2-4 times a week, 5-6 times a week, once or more daily*)	1339
Number of correct matches in round	The number of correct matches in the round.<br>Ordered categorical variable (n categories=10)	398
Number of days/weeks of moderate physical activity	Self-reported weekly moderate physical activity of 10 minutes or more (these are activities like carrying light loads, cycling at normal pace and include activities for work, leisure, travel and around the house but do not include walking)<br>Ordered categorical variable (n of categories=8)	884
Number of days of vigorous physical activity	Self-reported weekly vigorous activity of 10 minutes or more (these are activities that make you sweat or breathe hard such as fast cycling, aerobics, heavy lifting and include activities for work, leisure, travel and around the house).<br>Ordered categorical variable (n of categories=8)	904
Oily fish intake	Self-reported average oily fish intake (e.g. sardines, salmon, mackerel, herring) considering intake over the last year.<br>Ordered categorical variable (*Never, less than once a week, once a week, 2-4 times a week, 5-6 times a week, once or more daily*)	1329
Pork intake	Self-reported average pork intake (not including processed meats such as bacon or ham) considering intake over the last year.<br>Ordered categorical variable (*Never, less than once a week, once a week, 2-4 times a week, 5-6 times a week, once or more daily*)	1389
Salt added to food	Self-reported addition of salt to food (not including salt used in cooking) considering intake over the last year.<br>Ordered categorical variable (*Never/rarely, sometimes, usually, always*)	1478
Saturated fat intake	Estimated saturated fat intake, based on food and beverage consumption yesterday, excluding any supplements.	100006
Sleeplessness/insomnia	Self-reported sleeplessness/insomnia<br>Ordered categorical variable (*Never/rarely, sometimes, usually*)	1200

Usual walking pace	Self-reported walking pace, ordered categorical variable (*slow pace, steady average pace, and brisk pace*).<br>Slow pace is defined as less than 3 miles per hour.<br>Steady average pace is defined as between 3-4 miles per hour.<br>Fast pace is defined as more than 4 miles per hour.	924
Variation in diet	Self-reported weekly variation in diet. Ordered categorical variable (*Never/rarely, sometimes, often*)	1548
Frequency of walking for pleasure in the last 4 weeks	Self-reported frequency of walking for pleasure in last 4 weeks<br>Ordered categorical variable (Once in the last 4 weeks, 2-3 times in the last 4 weeks, once a week, 2-3 times a week,	971
Water intake	Self-reported daily water intake (glasses) considering intake over the last year. Ordered categorical variable (n categories=3)	1528

Risk factors implicated in Alzheimer’s disease in previous research

We selected four factors from the Global Burden of Disease Study (high BMI, high fasting plasma glucose, smoking, and a high intake of sugar-sweetened beverages) that contributed to metrics for deaths, prevalence, years of life lost, years of life lived with disability, and disability-adjusted life-years due to Alzheimer’s disease. The review identified the following as potentially modifiable risk factors for dementia; less education, midlife hypertension, obesity and hearing loss, as well as later life smoking, depression, physical inactivity, social isolation, and diabetes. Furthermore, a meta-analysis of case-control and population-based studies showed that rheumatoid arthritis is associated with lower incidence of Alzheimer’s disease. [19] The relationship between Alzheimer’s disease and rheumatoid arthritis has been studied before using genetic-based methods such as Mendelian randomization, [19] hence it is not examined here. We examined the use of methotrexate (anti-inflammatory drug for rheumatoid arthritis) due to observational studies [20,21] suggesting anti-inflammatory medicines for rheumatoid arthritis reduces risk of Alzheimer’s disease [20]. At the time of the analysis, plasma glucose was not available and was not investigated.

Replication

The Trøndelag Health Study (HUNT) is a population-based study of ~125,000 participants, which invited the entire adult (≥20 years) population of Trøndelag. Adults were invited for questionnaires, interviews, clinical examinations, laboratory measurements, and/or storage of biological samples in at least one of four study rounds so far, including HUNT1 (1984 to 1986, N=75,027, 86.8% of invited), HUNT2 (1995 to 1997, N=65,402, 69.7% of invited), HUNT3 (2006 to 2008, N=50,663, 54.0% of invited), and HUNT4 (2017 to 2019, N=56,042, 54.0% of invited). [22–24]

The current analysis includes genetic data from ~90% (N=71,860) of participants from HUNT2 and HUNT3 who were genotyped by genome-wide SNP arrays in 2015. [25,26] The genotyping and quality control metrics have been described elsewhere [5]. In brief, one of three different Illumina HumanCoreExome arrays (HumanCoreExome12 v1.0,

HumanCoreExome12 v1.1 and UM HUNT Biobank v1.0) were used for genotyping the HUNT2 and HUNT3 samples[5]. Participants of European origin were previously defined by projecting principal components (PC) of HUNT participants into the Human Genome Diversity Project (HGDP) reference panel[27,28]. Only individuals of European ancestry were included in the study.

For the replication of Alzheimer disease polygenic risk score (PRS)-outcome associations in the HUNT, we followed up 33 outcomes that were found to be statistically significant in UK Biobank and available with sufficient numbers in HUNT.

Information on outcomes were retrieved from HUNT2, HUNT3, and HUNT4 (from participants who were genotyped from either HUNT2 or HUNT3) where information from the available latest round of HUNT was given priority during analysis (priority order: HUNT4, HUNT3, and HUNT2).

The measurements analysed from HUNT2, HUNT3, and HUNT4 were weight (kg), waist circumference (cm), hip circumference (cm), and body mass index (kg/m^2). Measurements of whole body fat mass (kg), body fat percentage (%), and fat free mass (kg) measured using bio-electrical impedance analysis, and blood monocytes count (10^9/L), blood monocytes count percentage (%), blood erythrocytes count (10^{12}/L), and haemoglobin concentration (g/dL) using a Sysmex XN-1000 instrument were analysed from in HUNT4. Finally, in HUNT2, HUNT3, and HUNT4, measurements of pulse rate and diastolic blood pressure which were measured using a automated measures (Dinamap instrument) were analysed. For each participant, the second and third measurements of pulse rate and diastolic blood pressure were taken one minute apart. For the analysis, if three measurements were available, then the average of last two measurements was used but if only two measurements were available, then the last reading was used.

From the questionnaire and interview, information analysed included questions on mother diabetes ever (HUNT2: no, yes), mother high blood pressure ever (HUNT2: no, yes), weight

reduction in at least 2 kg compared with 1 year ago (HUNT3: no, yes), medication acetylsalicylic acid (Albyl E 500mg, Aspirin, Globoid, Dispril) last month (HUNT3: no, yes), frequency of glass of drinking water (HUNT3: <1 per week, 1-6 per week, 1 per day, 2-3 per day, ≥4 per day), frequency of glass of milk (HUNT3: <1 per week, 1-6 per week, 1 per day, 2-3 per day, ≥4 per day (Proxy for the 'Never eats dairy variable' in UK Biobank)), frequency of low-fat fish (HUNT4: <1 per week, 1-3 per week, 4-6 per week, ≥7 per week), frequency of red meat (HUNT4: <1 per week, 1-3 per week, 4-6 per week, ≥7 per week), hours of night sleep on a regular weekday (HUNT4), use fat on bread (HUNT2 and HUNT3: no, yes), bread type (HUNT2 and HUNT3: whole grain bread, white bread), usually eat white bread (HUNT2 and HUNT3: no, yes), usually eat whole grain bread (HUNT2 and HUNT3: no, yes), cardiac angina pectoris ever (HUNT2 and HUNT3: no, yes), average hours of light physical activity per week in the last year (HUNT2 and HUNT3: none, <1 hour, 1-2 hours, ≥3 hours), average hours of vigorous physical activity per week in the last year (HUNT2 and HUNT3: none, <1 hour, 1-2 hours, ≥3 hours), frequency of high-fat fish (HUNT3 and HUNT4: <1 per week, 1-3 per week, 4-6 per week, ≥7 per week), insomnia night in last three months (HUNT3 and HUNT4: never/seldom, sometimes, several times a week), frequency of food vegeSupplementary Tables (HUNT3 and HUNT4: <1 per week, 1-3 per week, 4-6 per week, ≥7 per week), upper back or lumbar pain in last 12 months (HUNT2, HUNT3, and HUNT4: no, yes)

Statistical analysis

For the replication analysis in the HUNT study, we used standardized weighted polygenic risk scores of the Alzheimer's disease SNPs in Supplementary Table 1 In the HUNT study, there is a large amount of relatedness between participants[5] therefore, to avoid the need to exclude related participants we used a method that accounts for the genetic relatedness using restricted maximum likelihood (REML) approach[8]. We fit a linear mixed model where a genome-wide genetic relationship matrix (GRM) was used to account for the relatedness across the sample[8]. The models were adjusted for age, sex and study participation round (if

the outcome was measured in multiple rounds of HUNT study), batch, and 10 principal components (PCs). Analyses were performed using R 4.0.3 (http://www.r-project.org) and GCTA software (gcta_1.93.3beta2). [29]

Follow-up using MR

Exposure GWASs were based on summary statistics from UK Biobank and were performed with the BOLT-LMM software package[30] using a published pipeline, [5] described in detail in the Supplementary material unless there was a larger published GWAS. For GWAS (GIANT was used for body mass index, hip and waist circumference), we used summary statistics from the GIANT consortium as it had larger sample sizes than UK Biobank alone. [31,32] Genome-wide association analysis (GWAS) was conducted using linear mixed model (LMM) association method as implemented in BOLT-LMM (v2.3). [30] To model population structure in the sample we used 143,006 directly genotyped SNPs, obtained after filtering on MAF > 0.01; genotyping rate > 0.015; Hardy-Weinberg equilibrium p-value < 0.0001 and LD pruning to an r2 threshold of 0.1 using PLINK v2.00. Genotype array and sex were adjusted for in the model. BOLT-LMM association statistics are on the linear scale. As such, test statistics (betas and their corresponding standard errors) were transformed to log odds ratios and their corresponding 95% confidence intervals on the liability scale using a Taylor transformation expansion series. [33]

Supplementary Table 4: Sample size of cases and controls for binary phenotypes used in Mendelian randomization analysis

Description	Cases	Controls	mu	Field ID
Diagnosis of angina	14,828	447,052	0.03	6150#2
Diagnosis of atherosclerotic heart disease	12,171	450,839	0.03	41202#I251
Diagnosis of cholecystectomy	18,319	444,614	0.04	20004#1455
Diagnosis of heart attack	10,693	451,187	0.02	6150#1
Diagnosis of heart attack/myocardial infarction (self-reported)	10,616	452,317	0.02	20002#1075
Diagnosis of high cholesterol (self-reported)	56,753	406,180	0.12	20002#1473
Diagnosis of intussusception	53	462,957	$1.14x10^{-4}$	41202#K561
Diagnosis of pure hypercholesterolaemia	22,622	440,388	0.05	41204#E780
Father still alive	103,919	346,414	0.23	1797
Hyperopia (left eye) – derived variable	34,518	79,367	0.30	5085
Hyperopia (right eye) – derived variable	33,361	80,897	0.29	5084
Injection of thrombin NEC	15	462,995	3.24E-05	41200#X304
Maternal history of diabetes	40,091	383,801	0.09	20110#9
Maternal history of high blood pressure	130,948	295,443	0.31	20110#8
Mother still alive	180,472	274,527	0.40	1835
Myopia (left eye) – derived variable	48,092	65,793	0.42	5085
Myopia (right eye) – derived variable	64,985	49,273	0.43	5084
Never eats dairy	10,366	450,680	0.02	6144#2
Never eats eggs or food containing eggs	12,077	448,969	0.26	6144#1
Paternal history of chronic bronchitis/emphysema	46,263	356,126	0.11	20107#6
Presence of aortocoronary bypass	3,358	459,652	0.007	41204#Z951
Use of aspirin	61,702	401,231	0.13	20003#1140868226
Use of atenolol	17,884	445,049	0.039	20003#1140866738
Use of ezetimibe	2701	460232	0.006	20003#1141192736
Use of fluconazole	55	462878	0.0001	20003#1140874272
Use of lipitor	3349	459584	0.007	20003#1141146138

Use of rosuvastatin	2870	460063	0.006	20003#1141192410
Use of simvastatin	52427	410506	0.11	20003#1140861958
Uses flora pro-active/benecol	33,414	185,575	0.18	2654-7#2
Uses white bread	106,619	298,546	0.36	1448-3#1
Never/rarely uses spreadable butter	153,140	265,971	0.58	1428-3#1
Wheeze/whistling	95131	358828	0.21	2316

Supplementary Note 1: Phenome-wide association study

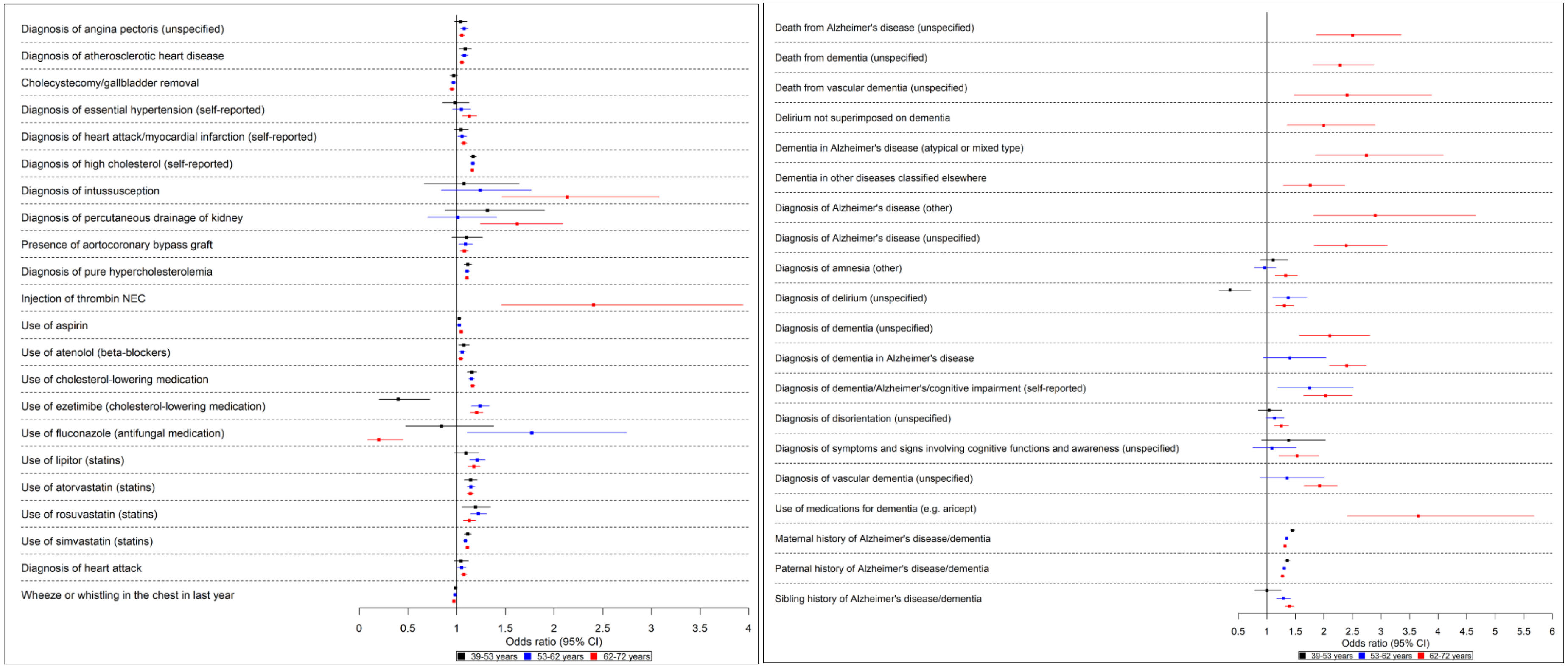

Supplementary Figure 3. Forest plots showing effect estimates for the association between polygenic risk score including the *APOE* region, general medical history and dementia-associated medical history by age tertile. Legends in each graph indicate age tertiles. Effect estimates represent an SD change in the phenotype per 1 unit increase in the standardized polygenic risk score for Alzheimer's disease. Error bars represent 95% confidence intervals. Each tertile consists of 111,656 participants and the exact sample size for each phenotype are in the Supplementary Data file.

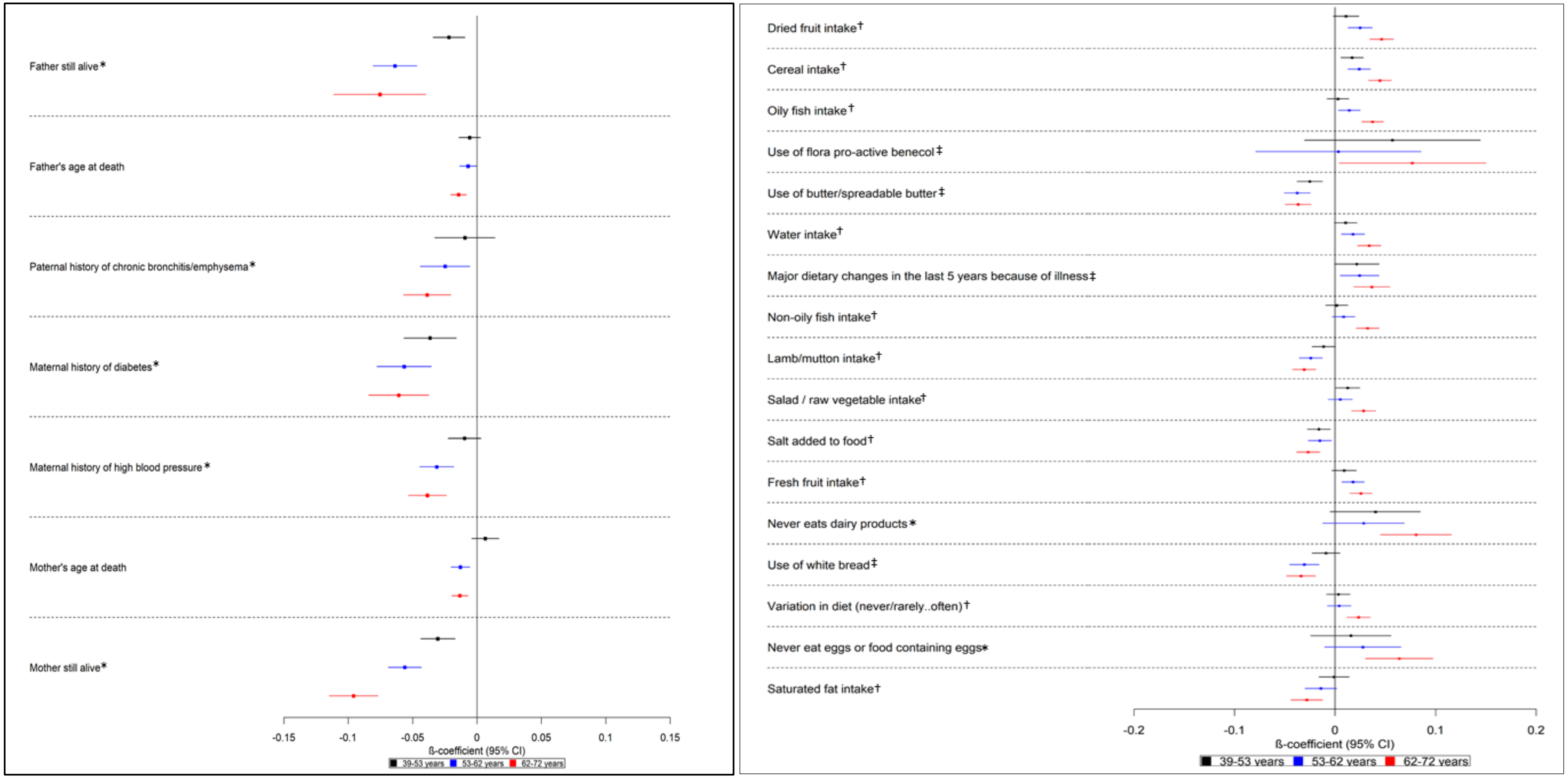

Supplementary Figure 4. Forest plots showing effect estimates for the association between polygenic risk score including the *APOE* region, family history and dietary choices by age tertile. Legends in each graph indicate age tertiles. Effect estimates represent an SD change in the phenotype per 1 unit increase in the standardized polygenic risk score for Alzheimer's disease. Error bars represent 95% confidence intervals. *Effect estimates were derived from binary logistic models and are on the log odds scale. ‡Effect estimates were derived from multinomial logistic models and are on the log odds scale. Each tertile consists of 111,656 participants and the exact sample size for each phenotype are in the Supplementary Data file.

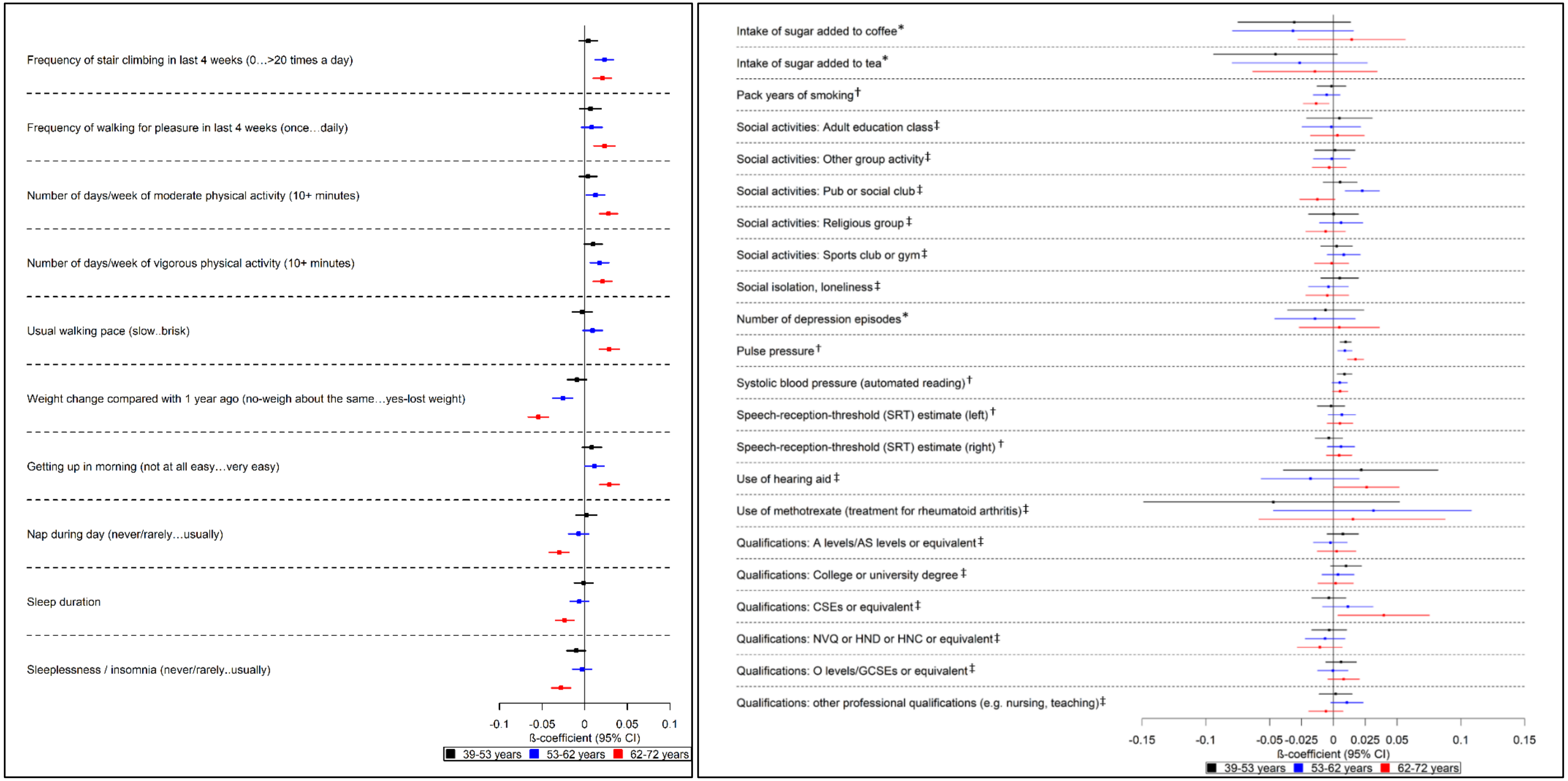

Supplementary Figure 5. Forest plot showing the effect estimates for the association between the polygenic score for Alzheimer's disease (including the *APOE* region), lifestyle, and previously implicated risk factors for Alzheimer's disease (that did not pass the corrected p-value threshold for multiple testing). Legends in each graph indicate age tertiles. Effect estimates represent an SD change in the phenotype per 1 unit increase in the standardized polygenic risk score for Alzheimer's disease. Error bars represent 95% confidence intervals. Each tertile consists of 111,656 participants and the exact sample size for each phenotype are in the Supplementary Data file. All effect estimates in the left panel were derived from ordered logistic regression models. Legends in each graph indicate age tertiles. Effect estimates are shown by box markers and confidence bands represent 95% confidence intervals. All effect estimates in the left panel were

derived from ordered logistic regression models. *Effect estimates were derived from ordered logistic models and effect estimates are on the log odds scale. †Effect estimates were derived from linear regression models and are in standard deviations. ‡Effect estimates were derived from binary logistic regression models and are on the log odds scale.

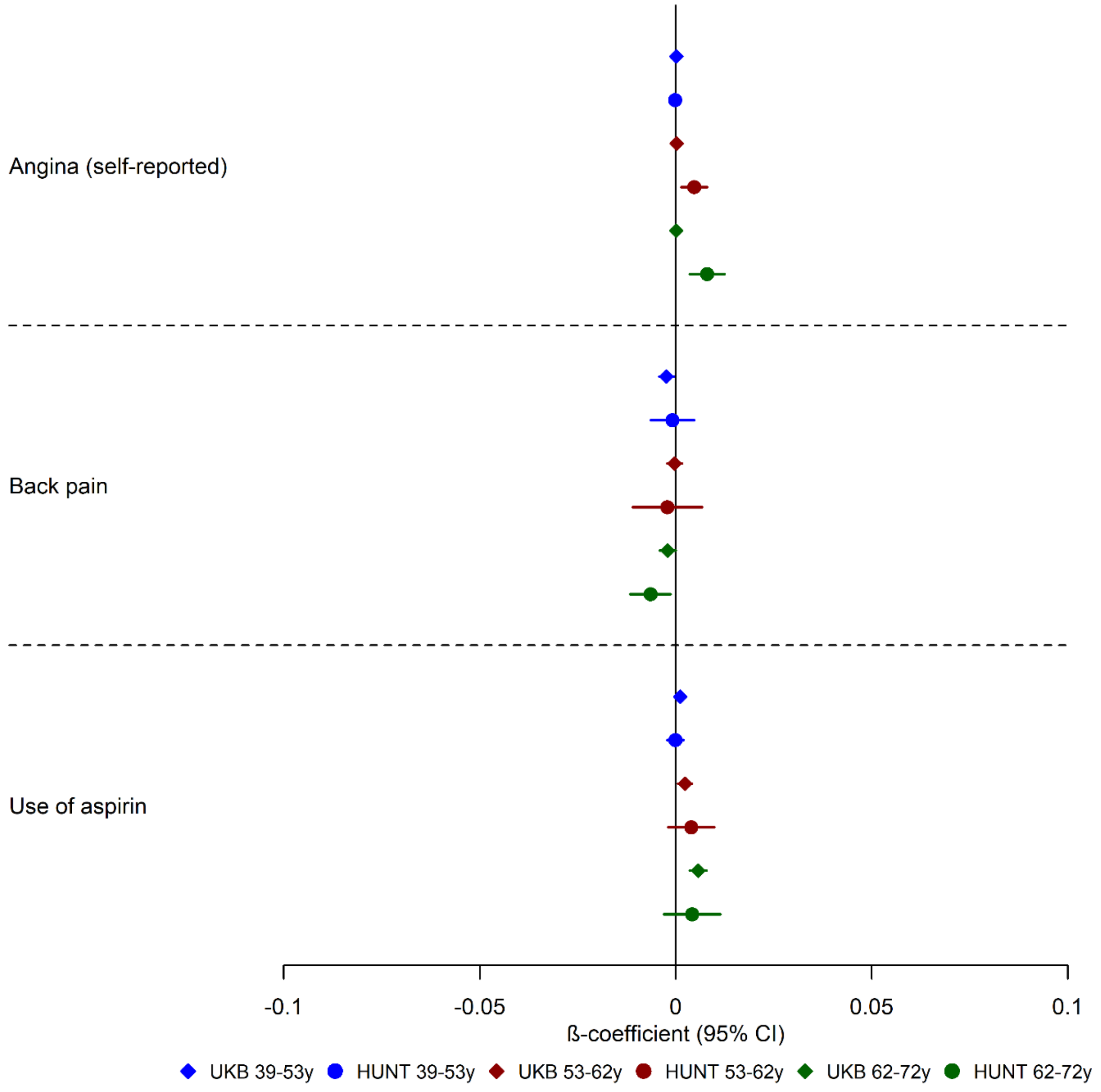

Supplementary Figure 6. Forest plot showing the age-stratified effect estimates for the association between the polygenic score for Alzheimer's disease (including the APOE region), and participant medical history in UK Biobank (diamond markers) and HUNT (replication sample, circle markers). Legends at the bottom of each graph indicate age tertiles in both cohorts. Effect estimates represent an SD change in the phenotype per 1 unit increase in the standardized polygenic risk score for Alzheimer's disease. Error bars represent 95% confidence intervals. The colours represent: blue, the youngest age tertile (39-53 years); red; middle age tertile (53-62 years); green, the oldest age tertile (62-72 years). The UK Biobank analyses included 111,418-111,638 participants in each age tertile. The HUNT analyses included 7,754-33,270 participants in each age tertile. The confidence intervals are smaller in UK Biobank due to the larger sample size of the cohort, compared to HUNT. Additionally, UK Biobank variables were converted to the risk difference scale to be directly comparable to effect estimates from the HUNT study.

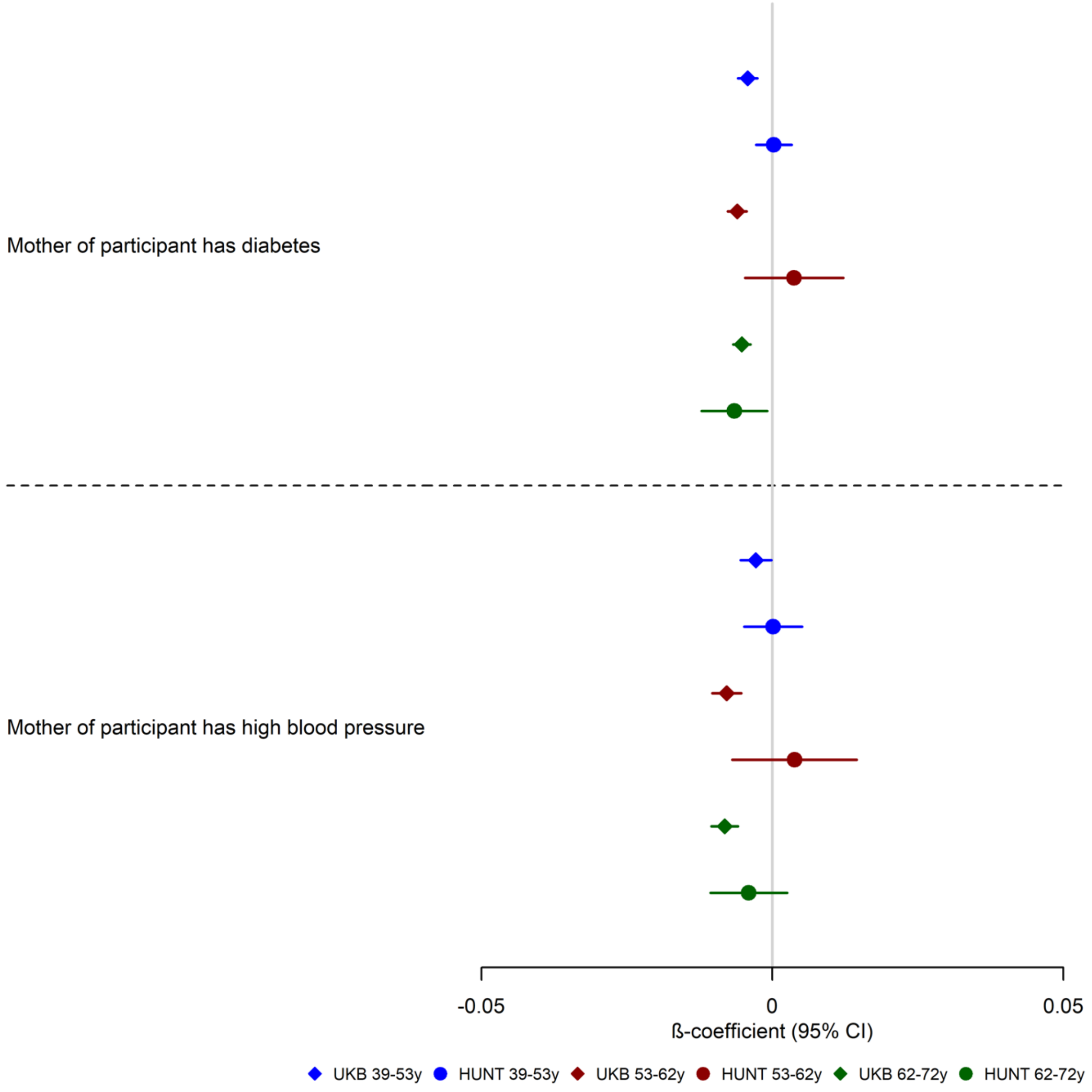

Supplementary Figure 7. Forest plot showing the age-stratified effect estimates for the association between the polygenic score for Alzheimer's disease (including the APOE region) and family medical history in UK Biobank (diamond markers) and HUNT (replication sample, circle markers). Legends at the bottom of each graph indicate age tertiles in both cohorts. Effect estimates represent an SD change in the phenotype per 1 unit increase in the standardized polygenic risk score for Alzheimer's disease. Error bars represent 95% confidence intervals. The colours represent: blue, the youngest age tertile (39-53 years); red; middle age tertile (53-62 years); green, the oldest age tertile (62-72 years). The UK Biobank analyses included 100,221-104,801 participants in each age tertile. The HUNT analyses included 6,569-27,740 participants in each age tertile. The confidence intervals are smaller in UK Biobank due to the larger sample size of the cohort, compared to HUNT. Additionally, UK Biobank variables were converted to the risk difference scale to be directly comparable to effect estimates from the HUNT study.

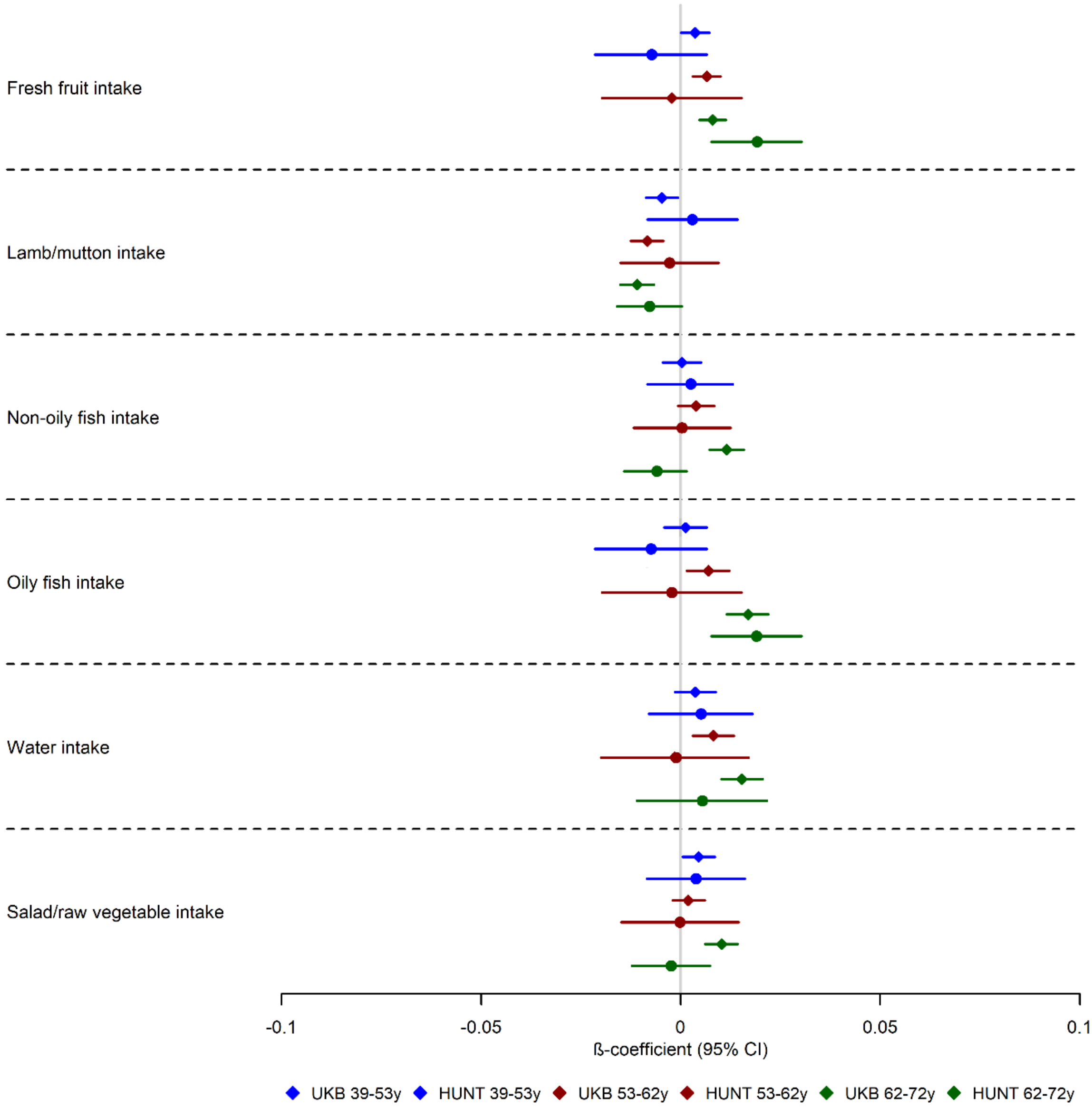

Supplementary Figure 8. Forest plot showing the age-stratified effect estimates for the association between the polygenic score for Alzheimer's disease (including the APOE region) and dietary habits in UK Biobank (diamond markers) and HUNT (replication sample, circle markers). Legends at the bottom of each graph indicate age tertiles in both cohorts. Effect estimates represent an SD change in the phenotype per 1 unit increase in the standardized polygenic risk score for Alzheimer's disease. Error bars represent 95% confidence intervals. The colours represent: blue, the youngest age tertile (39-53 years); red; middle age tertile (53-62 years); green, the oldest age tertile (62-72 years). The UK Biobank analyses included 102,012-111,313 participants in each age tertile. The HUNT analyses included 7,545-25,575 participants in each age tertile. The confidence intervals are smaller in UK Biobank due to the larger sample size of the cohort, compared to HUNT. Additionally, UK Biobank variables were converted to the risk difference scale to be directly comparable to effect estimates from the HUNT study.

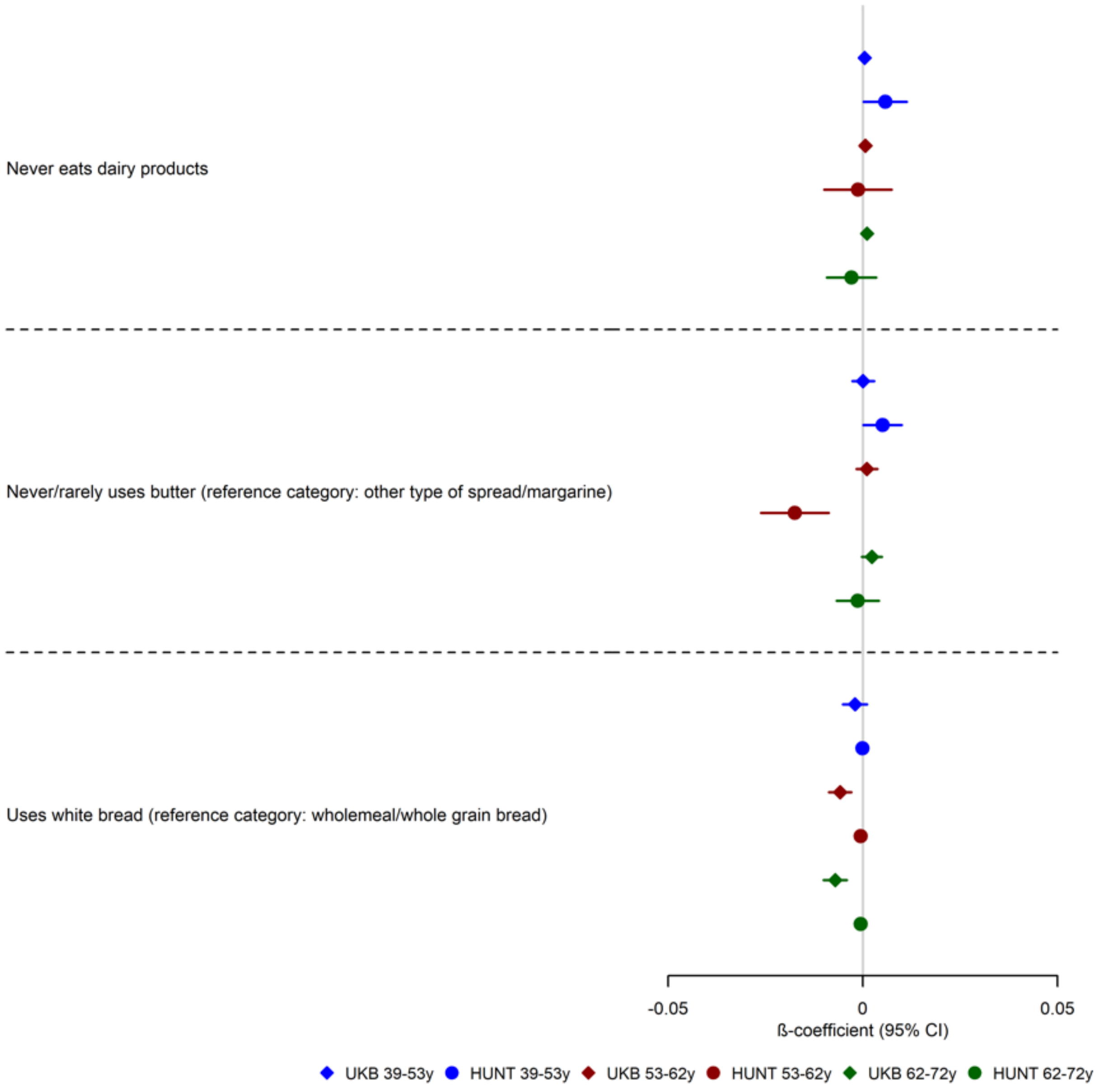

Supplementary Figure 9. Forest plot showing the age-stratified effect estimates for the association between the polygenic score for Alzheimer's disease (including the *APOE* region) and dietary habits in UK Biobank (diamond markers) and HUNT (replication sample, circle markers). Legends at the bottom of each graph indicate age tertiles in both cohorts. Effect estimates represent an SD change in the phenotype per 1 unit increase in the standardized polygenic risk score for Alzheimer's disease. Error bars represent 95% confidence intervals. The colours represent: blue, the youngest age tertile (39-53 years); red; middle age tertile (53-62 years); green, the oldest age tertile (62-72 years). The UK Biobank analyses included 107,382-111,472 participants in each age tertile. The HUNT analyses include 9,801-27,854 participants in each age tertile. The confidence intervals are smaller in UK Biobank due to the larger sample size of the cohort, compared to HUNT. Additionally, UK Biobank variables were converted to the risk difference scale to be directly comparable to effect estimates from the HUNT study.

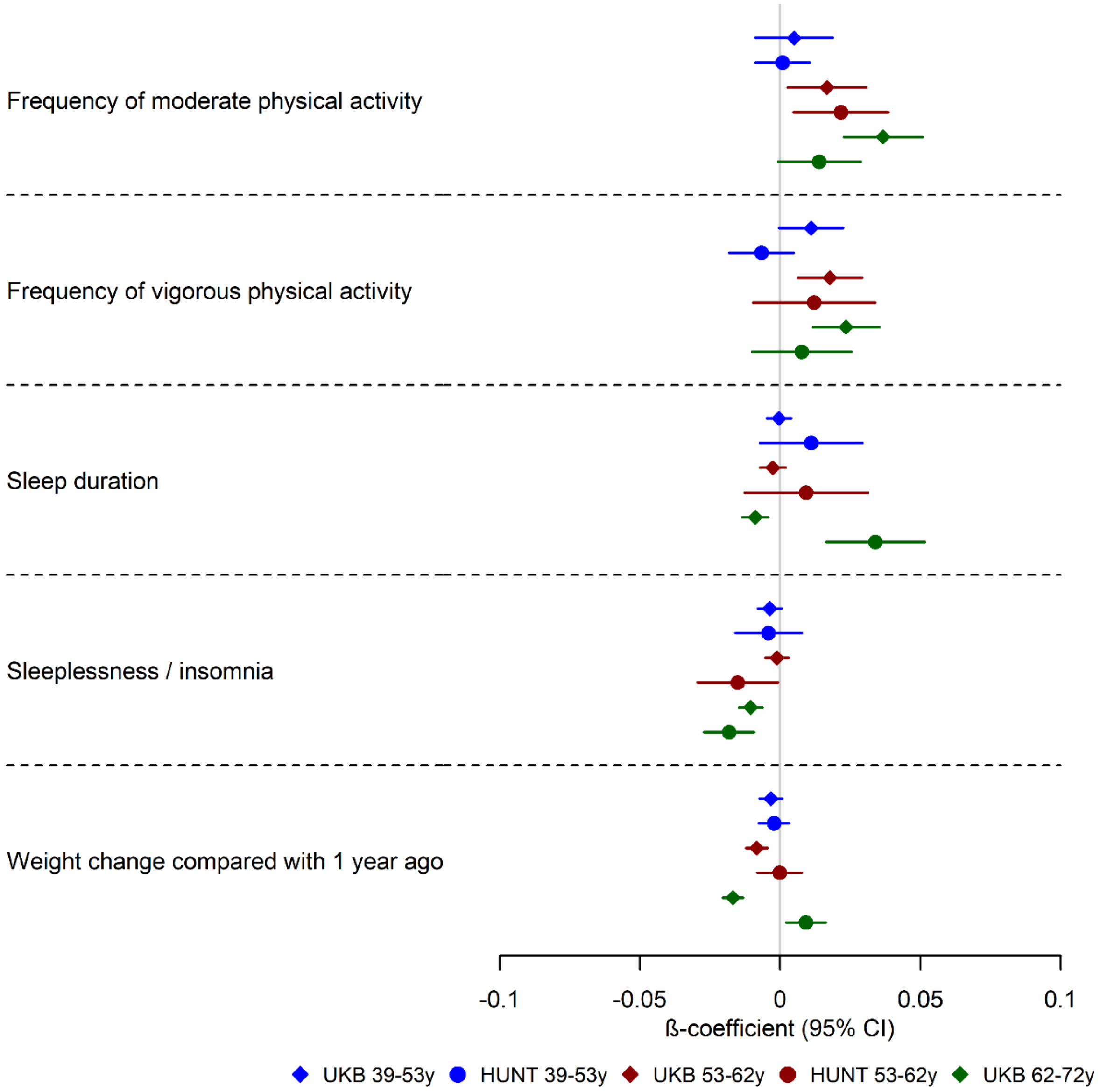

Supplementary Figure 10. Forest plot showing the age-stratified effect estimates for the association between the polygenic score for Alzheimer's disease (including the *APOE* region) and lifestyle in UK Biobank (diamond markers) and HUNT (replication sample, circle markers). Legends at the bottom of each graph indicate age tertiles in both cohorts. Effect estimates represent an SD change in the phenotype per 1 unit increase in the standardized polygenic risk score for Alzheimer's disease. Error bars represent 95% confidence intervals. The colours represent: blue, the youngest age tertile (39-53 years); red; middle age tertile (53-62 years); green, the oldest age tertile (62-72 years). The UK Biobank analyses included 104,395-111,601 participants in each age tertile. The HUNT analyses included 7,468-29,976 participants in each age tertile. The confidence intervals are smaller in UK Biobank due to the larger sample size of the cohort, compared to HUNT. Additionally, UK Biobank variables were converted to the risk difference scale to be directly comparable to effect estimates from the HUNT study.

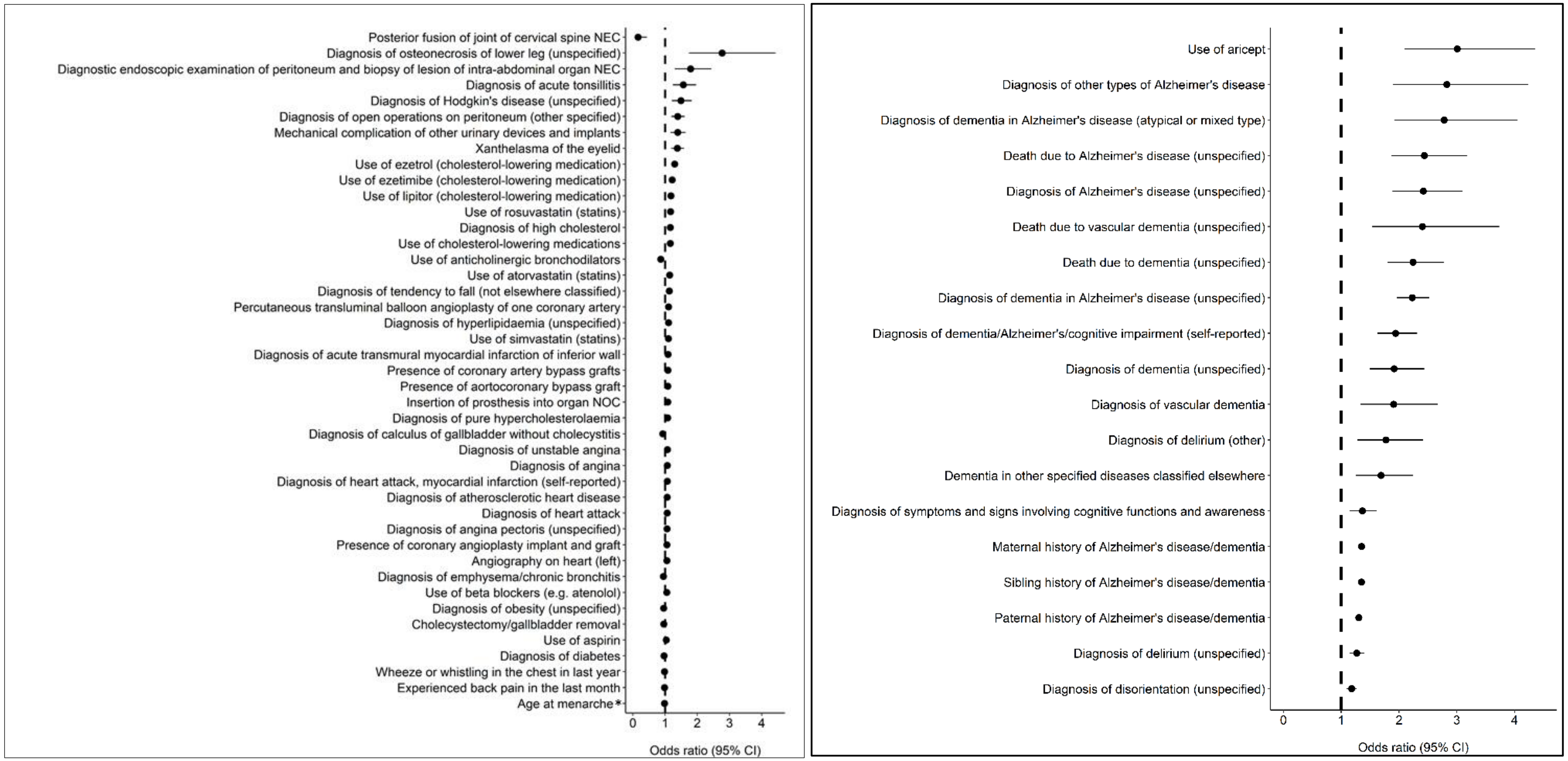

Supplementary Figure 11. Forest plots showing effect estimates for the association between polygenic risk score including SNPs in the *APOE* region, medical history and dementia-associated medical history in the entire UK Biobank sample. Forest plot (left) shows the effect estimates related to the medical history of the participants (diagnoses, medicines, operations). Forest plot (right) shows effect estimates of dementia-associated medical history. These analyses included 334,968 participants. Effect estimates represent an SD change in the phenotype per 1 unit increase in the standardized polygenic risk score for Alzheimer's disease. Error bars represent 95% confidence intervals.

Abbreviations: NEC, not elsewhere classified; NOC, not otherwise classified. *Effect estimates were derived from an ordered logistic model.

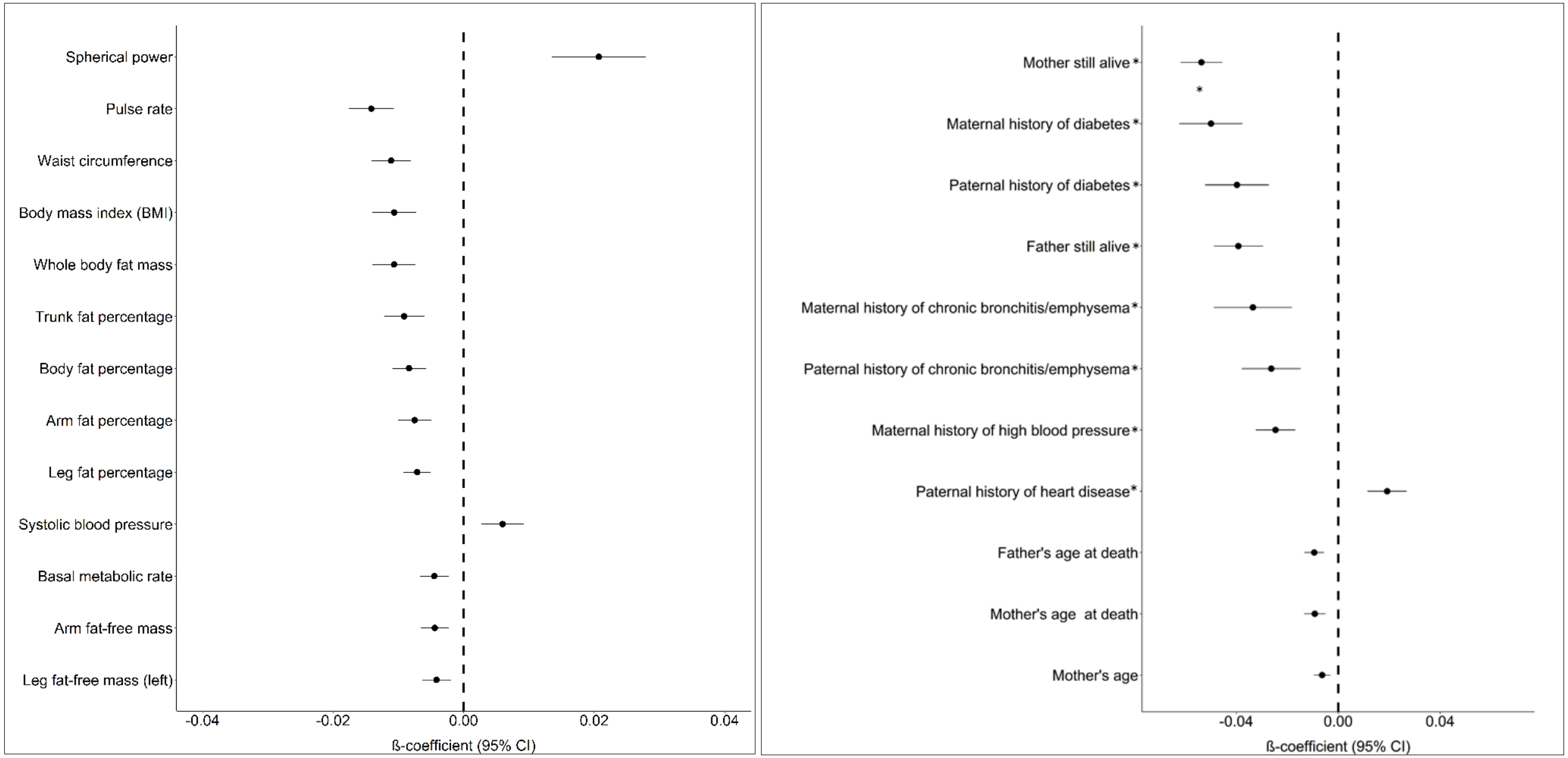

Supplementary Figure 12. Forest plots showing effect estimates for the association between polygenic risk score including SNPs in the *APOE* region, parental health factors, and physical measures in the entire UK Biobank sample. Forest plot (left) shows the effect estimates related to parental health factors of the participants. Forest plot (right) shows effect estimates of outcomes related to physical measures of the participants. Effect estimates represent an SD change in the phenotype per 1 unit increase in the standardized polygenic risk score for Alzheimer's disease. Error bars represent 95% confidence intervals. These analyses included 334,968 participants.

*Effect estimates were derived from binary logistic models and are on the log odds scale.

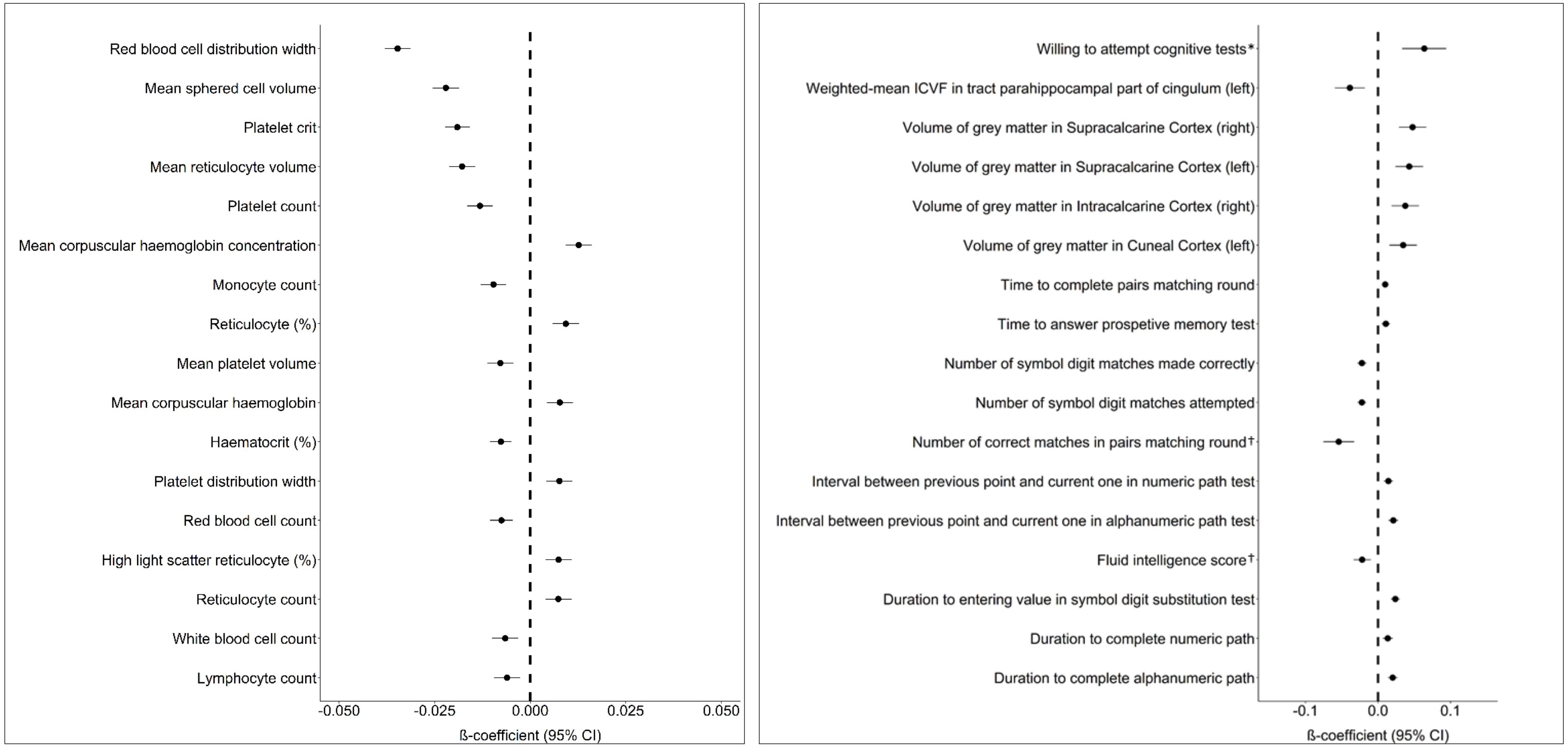

Supplementary Figure 13. Forest plots showing effect estimates for the association between polygenic risk score including SNPs in the *APOE* region, biological measures, brain-related and cognitive test measures in the entire UK Biobank sample. Forest plot (left) shows the effect estimates related to brain-related and cognitive test measures. Forest plot (right) shows effect estimates related to biological sample measures. These analyses included 334,968 participants. Effect estimates represent an SD change in the phenotype per 1 unit increase in the standardized polygenic risk score for Alzheimer’s disease. Error bars represent 95% confidence intervals. These analyses included 334,968 participants.

*Effect estimates are from binary logistic models and are on the log odds scale. †Effect estimates are from ordered logistic models and are on the log odds scale.

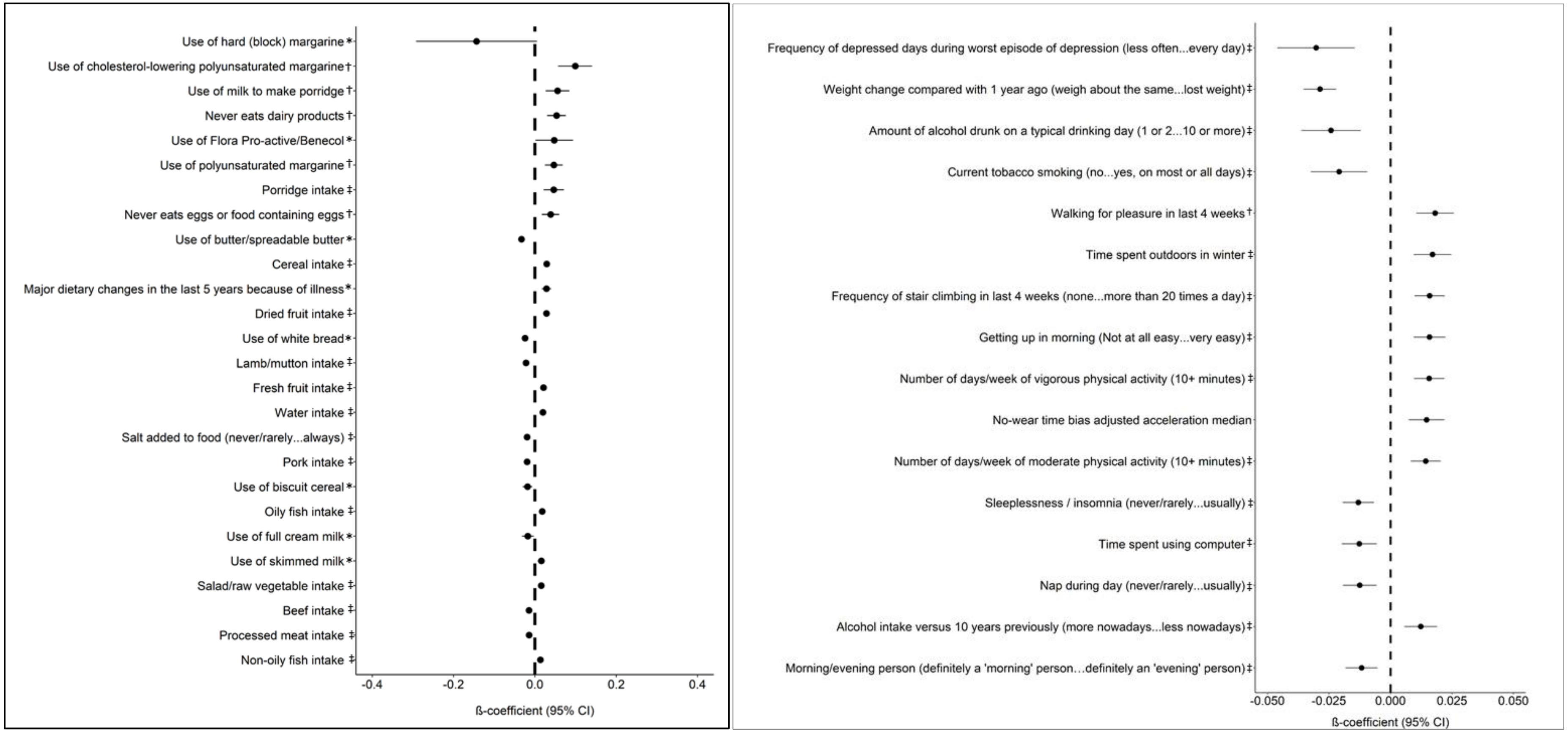

Supplementary Figure 14. Forest plots showing effect estimates for the association between polygenic risk score including SNPs in the *APOE* region, dietary choices, and lifestyle in the entire UK Biobank sample. Forest plot (left) shows the effect estimates related to the dietary choices of participants. Forest plot (right) shows the effect estimates related to lifestyle. Effect estimates represent an SD change in the phenotype per 1 unit increase in the standardized polygenic risk score for Alzheimer's disease. Error bars represent 95% confidence intervals. These analyses included 334,968
participants.
*Effect estimates were derived from multinomial logistic models and are on the log odds scale.†Effect estimates were derived from binary logistic models and are on the log odds scale. ‡Effect estimates were derived from ordered logistic models and are on the logs scale for forest plot displaying lifestyle measures. Reference categories for multinomial logistic models are as follows: use of hard (block) margarine -

Polyunsaturated/sunflower; use of Flora Pro-active or Benecol – polyunsaturated/sunflower oil-based); use of white bread – wholemeal or wholegrain bread; use of full cream/skimmed milk – skimmed milk; use of biscuit cereal – oat cereal (e.g. Ready Brek); Major dietary changes in the last 5 years because of illness – no major dietary changes in the last 5 years; current smoker – never smoker

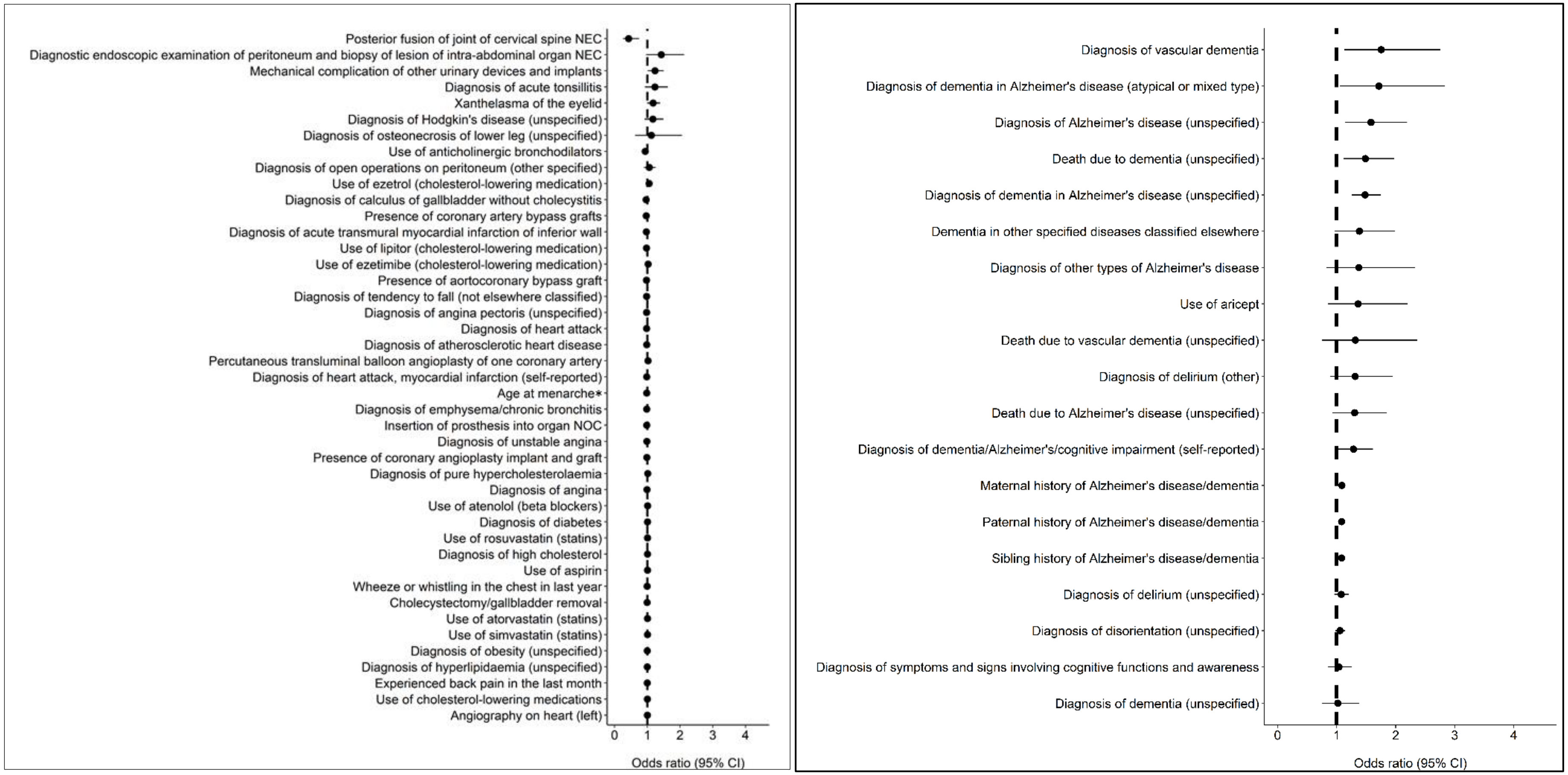

Supplementary Figure 15. Forest plots showing effect estimates for the association between polygenic risk score excluding *APOE* (for top hits when SNPs in the *APOE* region were included), medical history, and dementia-associated medical history in the entire UK Biobank sample. Forest plot (left) shows the effect estimates related to the medical history of the participants (diagnoses, medicines, operations). Forest plot (right) shows effect estimates of dementia-associated outcomes. Effect estimates represent an SD change in the phenotype per 1 unit increase in the standardized polygenic risk score for Alzheimer's disease. Error bars represent 95% confidence intervals.

These analyses included 334,968 participants. Abbreviations: NEC, not elsewhere classified; NOC, not otherwise classified.

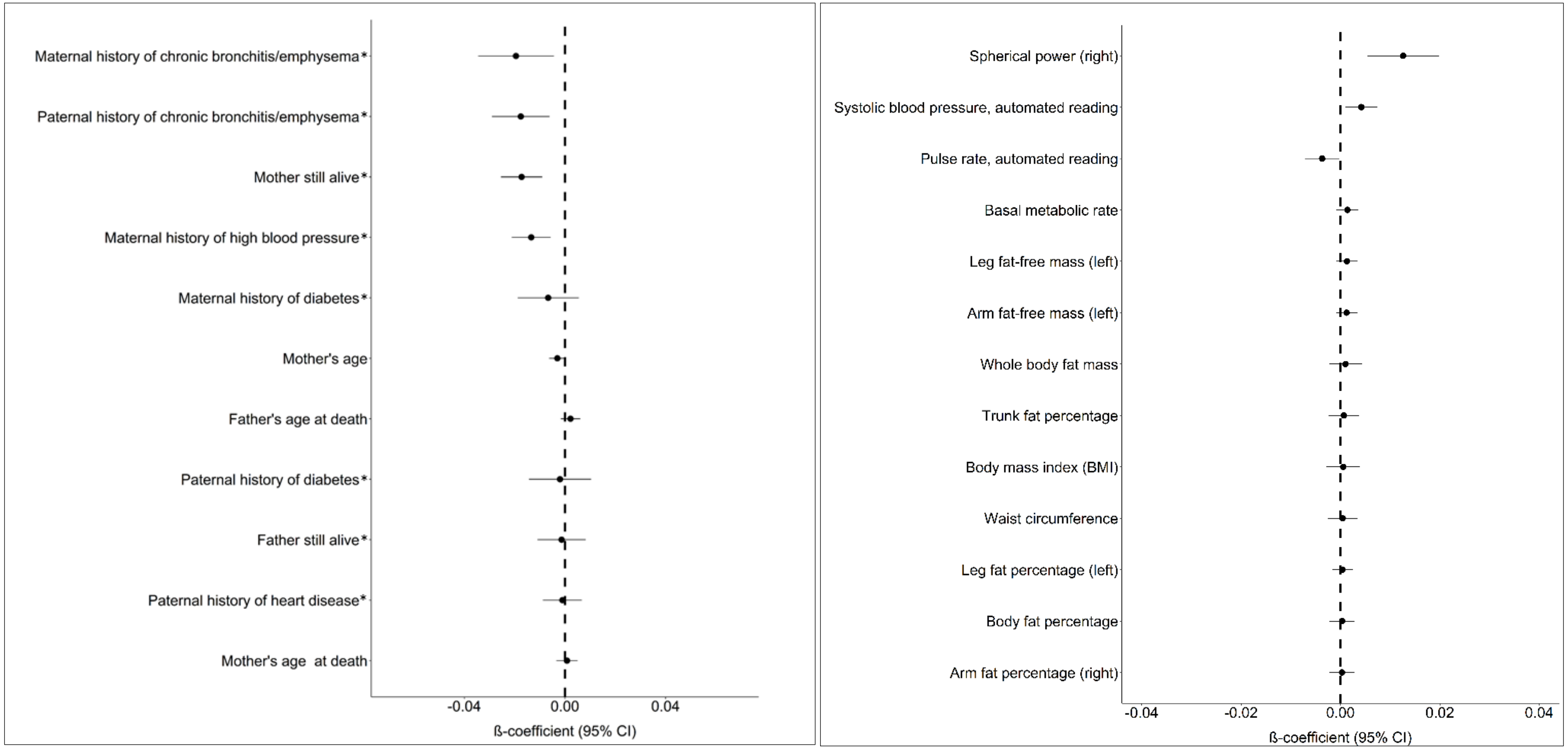

Supplementary Figure 16. Forest plots showing effect estimates for the association between polygenic risk score excluding *APOE* (for top hits when SNPs in the *APOE* region were included), parental health factors, and physical measures in the entire UK Biobank sample. Forest plot (left) shows the effect estimates related to parental health factors of the participants. Forest plot (right) shows effect estimates of outcomes related to physical measures of the participants. Effect estimates represent an SD change in the phenotype per 1 unit increase in the standardized polygenic risk score for Alzheimer's disease. Error bars represent 95% confidence intervals. These analyses included 334,968 participants.

*Effect estimates were derived from binary logistic models.

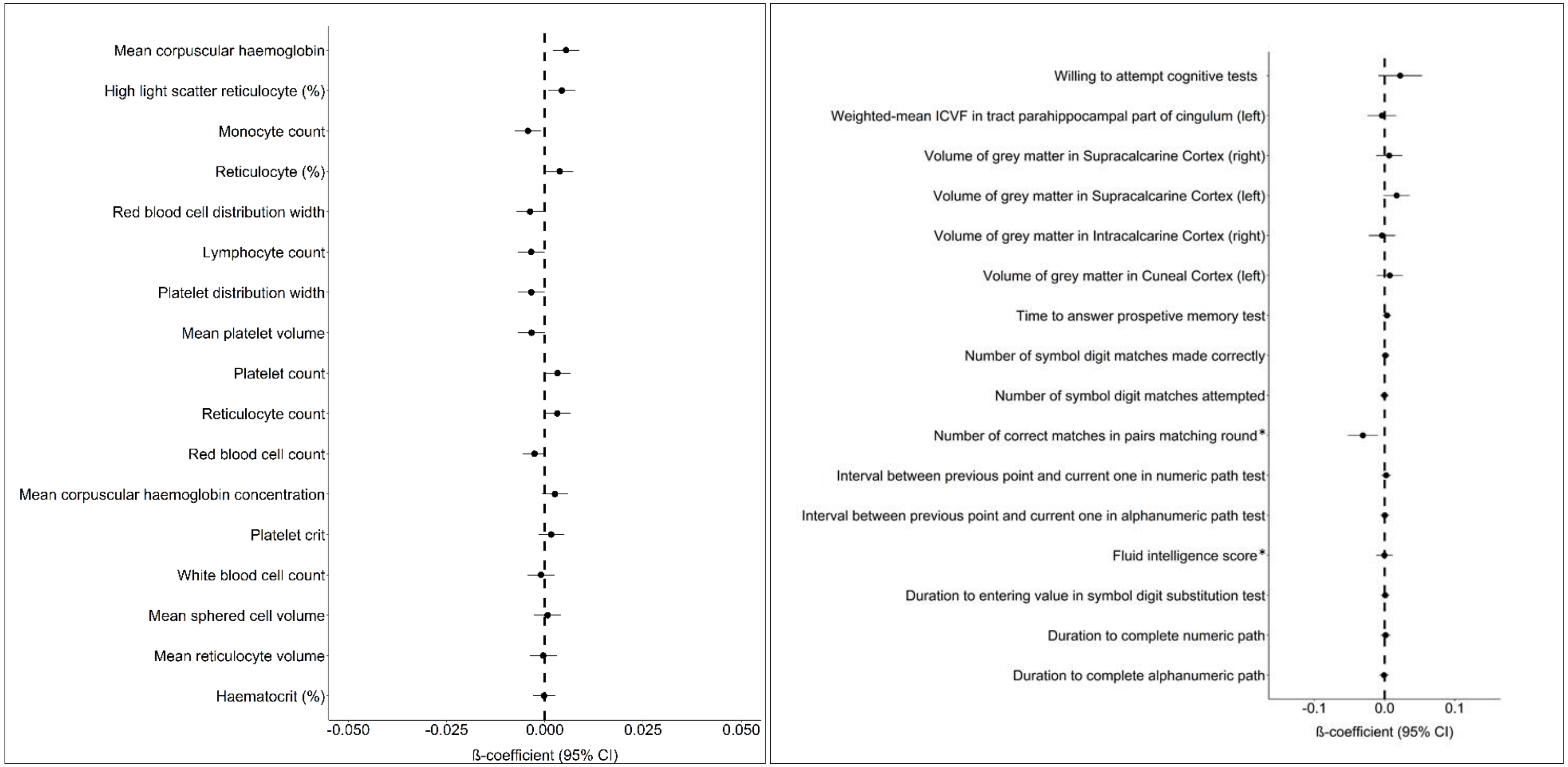

Supplementary Figure 17. Forest plots showing effect estimates for the association between polygenic risk score excluding *APOE* (for top hits when SNPs in the *APOE* region were included), biological, brain-related and cognitive test measures in the entire UK Biobank sample. Forest plot (left) shows the effect estimates related to biological sample measures. Forest plot (right) shows effect estimates related to brain-related and cognitive test measures. Effect estimates represent an SD change in the phenotype per 1 unit increase in the standardized polygenic risk score for Alzheimer's disease. Error bars represent 95% confidence intervals. These analyses included 334,968 participants.

*Effect estimates were derived from ordered logistic models and are on the log odds scale.

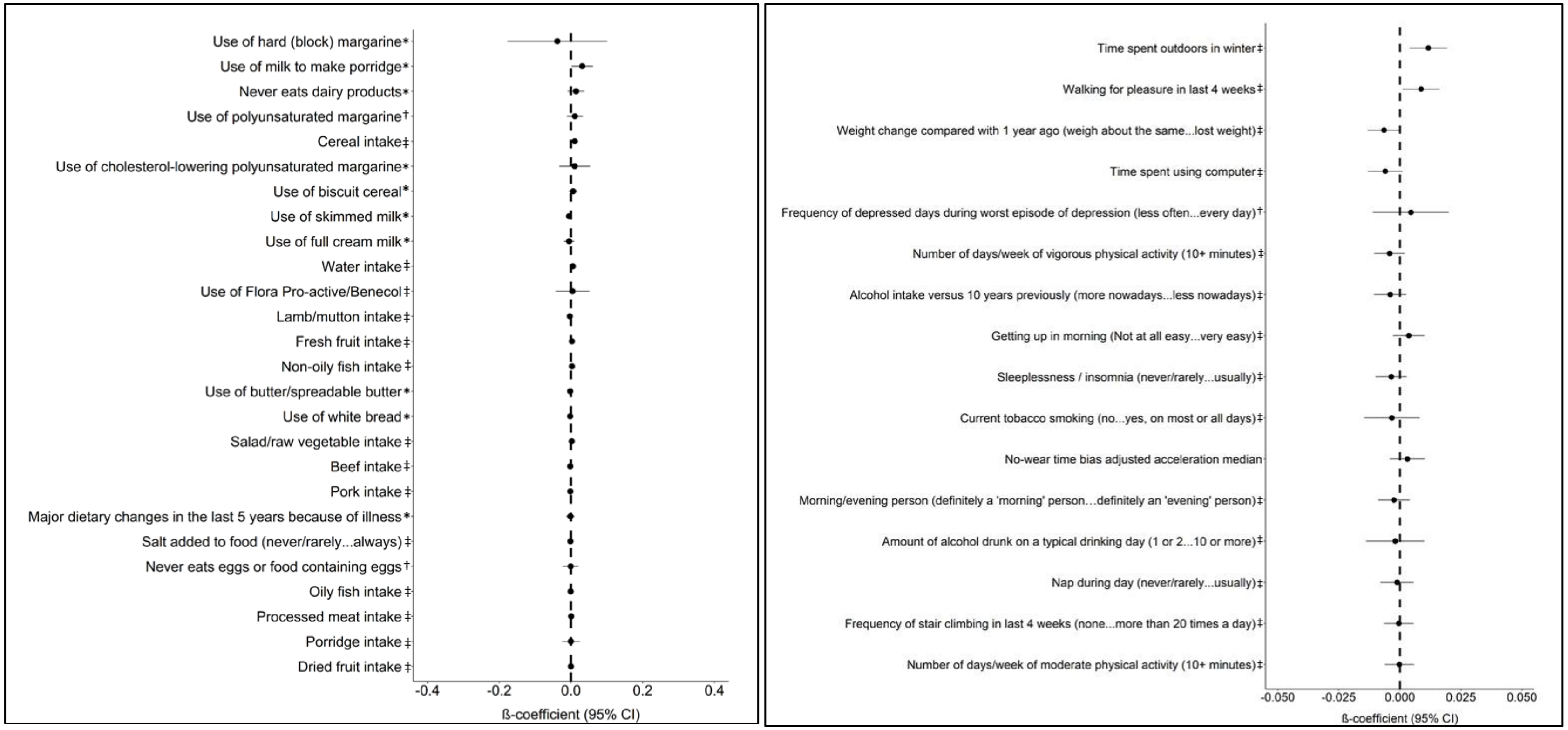

Supplementary Figure 18. Forest plots showing effect estimates for the association between polygenic risk score excluding *APOE* (for top hits when SNPs in the *APOE* region were included), dietary choices, and lifestyle measures in the entire UK Biobank sample. Forest plot (left) shows the effect estimates related to the dietary choices of participants. Forest plot (right) shows the effect estimates related to lifestyle. Effect estimates represent an SD change in the phenotype per 1 unit increase in the standardized polygenic risk score for Alzheimer's disease. Error bars represent 95% confidence intervals. These analyses included 334,968 participants.

Reference categories for multinomial logistic models are as follows: use of butter/spreadable butter - other type of spread/margarine; use of Flora Pro-active or Benecol -polyunsaturated/sunflower oil-based); †Effect estimates were derived from multinomial logistic models and are on log odds scale. *Effect estimates were derived from binary logistic models.

‡Effect estimates were derived from ordered logistic models.

Supplementary Table 5: Detailed description of multinomial outcome estimates for the PheWAS using the polygenic risk score including the APOE region

Variable	Reference	Comparison category	Sample size (Reference#comparison)	β-coefficient (95% CI)	P
Bread type (var 1448)	Wholemeal or wholegrain	White	187292#85025	-0.02 (-0.03, -0.02)	7.69×10^{-9}
		Brown	187292#38060	-0.003 (-0.01, 0.01)	0.55
		Other type of bread	187292#13044	-0.002 (-0.02, 0.02)	0.83
Cereal type (var 1468)	Oat cereal	Bran cereal	70455#47526	-0.004 (-0.02, 0.01)	0.48
		Muesli	70455#57151	-0.01 (-0.02, -0.001)	0.04
		Biscuit cereal	70455#50122	-0.02 (-0.03, -0.01)	0.0002
		Other (e.g. Frosties)	70455#54299	-0.03 (-0.04, -0.02)	1.06×10^{-8}
Major dietary changes (var 1538)	None	Because of illness	205929#35127	0.03 (0.02,0.04)	8.76×10^{-7}
		Because of other reasons	205929#93276	0.03 (0.03,0.04)	0
Milk type (var 1418)	Semi-skimmed	Never/rarely have milk	218496#10423	0.02 (-0.003, 0.04)	0.05
		Full cream	218496#20770	-0.02 (-0.03, -0.003)	0.02
		Skimmed	218496#69166	0.02 (0.01, 0.02)	0.0003
		Other type of milk	218496#3801	0.02 (-0.01, 0.05)	0.13
		Soya	218496#12152	0.02 (0.01, 0.04)	0.007
Non-butter spread type (var 2654)	Polyunsaturated/sunflower oil-based spread (eg: Flora)	Flora Pro-active or Benecol	60591#27028	0.07 (0.05,0.08)	0
		Soft (tub) margarine	60591#21790	-0.01 (-0.03,0.001)	0.06
		Hard (block) margarine	60591#199	-0.14 (-0.29, -0.005)	0.06
		Olive oil-based spread (eg. Bertoli)	60591#44013	0.005 (-0.01,0.02)	0.45
		Other low or reduced fat spread	60591#17718	0.02 (-0.01, -0.0003)	0.05
		Other type of spread/margarine	60591#6328	0.002 (-0.02,0.03)	0.87

Supplementary Table 6: Detailed description of multinomial outcome estimates for the PheWAS using the polygenic risk score excluding the *APOE* region

Variable	Reference	Comparison category	Sample size (Reference#comparison)	β-coefficient (95% CI)	P
Bread type (var 1448)	Wholemeal or wholegrain	White	187292#85025	-0.002 (-0.01, 0.006)	0.58
		Brown	187292#38060	-0.00002 (-0.01, 0.01)	0.99
		Other type of bread	187292#13044	0.01 (-0.003,0.03)	0.11
Cereal type (var 1468)	Oat cereal	Bran cereal	70455#47526	0.0041 (-0.01, 0.02)	0.52
		Muesli	70455#57151	-0.01 (-0.02,0.006)	0.37
		Biscuit cereal	70455#50122	0.006 (-0.01,0.02)	0.31
		Other (e.g. cornflakes,frosties)	70455#54299	0.0003 (-0.01, 0.01)	0.96
Major dietary changes (var 1538)	None	Because of illness	205929#35127	-0.002 (-0.01,0.01)	0.77
		Because of other reasons	205929#93276	-0.001 (-0.01, 0.01)	0.87
Milk type (var 1418)	Semi-skimmed	Never/rarely have milk	218496#10423	-0.01 (-0.03,0.008)	0.26
		Full cream	218496#20770	-0.01 (-0.02,0.01)	0.47
		Skimmed	218496#69166	-0.005 (-0.01, 0.003)	0.21
		Other type of milk	218496#3801	0.0003 (-0.03, 0.03)	0.99
		Soya	218496#12152	0.005 (-0.01,0.02)	0.59
Non-butter spread type (var 2654)	Polyunsaturated /sunflower oil-based spread (eg: Flora)	Flora Pro-active or Benecol	60591#27028	0.005 (-0.01, 0.02)	0.51
		Soft (tub) margarine	60591#21790	-0.003 (-0.02,0.01)	0.69
		Hard (block) margarine	60591#199	-0.04 (-0.18,0.10)	0.59
		Olive oil-based spread (eg. Bertoli)	60591#44013	0.003 (-0.01,0.02)	0.62
		Other low or reduced fat spread	60591#17718	0.02 (0.003,0.04)	0.02
		Other type of spread/margarine	60591#6328	0.002 (-0.02,0.03)	0.86

Supplementary Table 7: Detailed description of multinomial outcome estimates for the PheWAS using the polygenic risk score including the *APOE* region in tertile 1

Variable	Reference	Comparison category	Sample size (Reference#comparison)	β-coefficient (95% CI)	P
Bread type (var 1448)	Wholemeal or wholegrain	White	59117#31401	-0.01 (-0.02, 0.005)	0.20
		Brown	59117#12598	-0.004 (-0.02, 0.02)	0.67
		Other type of bread	59117#4266	0.02 (-0.01, 0.05)	0.20
Major dietary changes (var 1538)	No major dietary changes	Yes, because of illness	69407#8813	0.02 (-0.01, 0.04)	0.06
		Yes, because of other reasons	69407#33223	0.03 (0.01, 0.04)	8.21×10^{-5}
Non-butter spread type (var 2654)	Polyunsaturated/sunflower oil-based spread (eg: Flora)	Flora Pro-active or Benecol	20333#5462	0.04 (0.01,0.07)	0.006
		Soft (tub) margarine	20333#8059	-0.03 (-0.06, -0.01)	0.009
		Hard (block) margarine	20333#72	-0.29 (-0.55, -0.03)	0.03
		Olive oil-based spread(eg. Bertoli)	20333#14488	-0.01 (-0.03,0.01)	0.36
		Other low or reduced fat spread	20333#6056	0.0003 (-0.03,0.03)	0.98
		Other type of spread/margarine	20333#2495	-0.005 (-0.05,0.04)	0.82
Spread type (var 1428)	Other type of spread/margarine	Never/rarely use spread	57167#11519	-0.0001 (-0.02, 0.02)	0.99
		Butter/spreadable butter	57167#42259	-0.03 (-0.04, -0.01)	9.67×10^{-5}
		Flora Pro-active/Benecol	57167#480	0.06 (-0.03,0.14)	0.20

Supplementary Table 8: Detailed description of multinomial outcome estimates for the PheWAS using the polygenic risk score including the *APOE* region in tertile 2

Variable	Reference	Comparison category	Sample size (Reference#comparison)	β-coefficient (95% CI)	P
Bread type (var 1448)	Wholemeal or wholegrain	White	65470#25687	-0.03 (-0.05, -0.02)	4.66×10^{-5}
		Brown	65470#12233	-0.004 (-0.02,0.02)	0.69
		Other type of bread	65470#4257	-0.02 (-0.05,0.01)	0.20
Major dietary changes (var 1538)	No major dietary changes	Yes, because of illness	67046#12285	0.02 (0.005, 0.04)	0.01
		Yes, because of other reasons	67046#32111	0.03 (0.02,0.05)	7.62×10^{-7}
Non-butter spread type (var 2654)	Polyunsaturated/sunflower oil-based spread (eg: Flora)	Flora Pro-active or Benecol	20571#9458	0.07 (0.05,0.1)	1.46×10^{-9}
		Soft (tub) margarine	20571#6512	0.001 (-0.03,0.03)	0.93
		Hard (block) margarine	20571#52	-0.002 (-0.27, 0.27)	0.99
		Olive oil-based spread	20571#14738	0.01 (-0.01,0.03)	0.22
		Other low or reduced fat spread	20571#5973	0.01 (-0.02,0.04)	0.40
		Other type of spread/margarine	20571#2029	-0.01 (-0.06,0.03)	0.63
Spread type (var 1428)	Other type of spread/margarine	Never/rarely use spread	59494#11744	0.01 (-0.01,0.03)	0.41
		Butter/spreadable butter	59494#39619	-0.04 (-0.05, -0.02)	9.18×10^{-9}
		Flora Pro-active/Benecol	59494#570	0.003 (-0.08,0.09)	0.94

Supplementary Table 9: Detailed description of multinomial outcome estimates for the PheWAS using the polygenic risk score including the *APOE* region in tertile 3

Variable	Reference	Comparison category	N	β-coefficient (95% CI)	P
Bread type (var 1448)	Wholemeal or wholegrain	White	62705#27937	-0.03 (-0.05, -0.02)	$3.72x10^{-6}$
		Brown	62705#13229	-0.001 (-0.02,0.02)	0.91
		Other type of bread	62705#4521	-0.005 (-0.04,0.03)	0.75
Major dietary changes (var 1538)	No major dietary changes	Yes, because of illness	69476#14029	0.04 (0.02,0.05)	$3.89x10^{-8}$
		Yes, because of other reasons	69476#27942	0.04 (0.03,0.05)	$7.29x10^{-5}$
Non-butter spread type (var 2654)	Polyunsaturated/sunflower oil-based spread (eg: Flora)	Flora Pro-active or Benecol	19687#12108	0.08 (0.05,0.10)	$1.95x10^{-11}$
		Soft (tub) margarine	19687#7219	-0.007 (-0.03,0.02)	0.60
		Hard (block) margarine	19687#75	-0.12 (-0.36,0.13)	0.35
		Olive oil-based spread	19687#14787	0.01 (-0.01, 0.03)	0.44
		Other low or reduced fat spread	19687#5689	0.02 (-0.02, 0.07)	0.15
		Other type of spread/margarine	19687#1804	0.03 (-0.01,0.08)	0.32
Spread type (var 1428)	Other type of spread/margarine	Never/rarely use spread	61482#10561	0.02 (-0.001,0.04)	0.07
		Butter/spreadable butter	61482#38747	-0.04 (-0.05, -0.02)	$2.15x10^{-8}$
		Flora Pro-active/Benecol	61482#682	0.08 (0.004,0.15)	0.04

Supplementary Note 2: Follow-up using Mendelian randomization

Of the 165 phenotypes identified in the PheWAS and of the 12 previously implicated risk factors not identified in the PheWAS, we followed up 87 phenotypes, respectively, using two-sample bidirectional Mendelian randomization. We did not follow up 90 phenotypes identified in the PheWAS because of either low prevalence, no genetic instruments, or if they indicated own diagnosis or family history of Alzheimer's disease. For wheeze/whistling, we also examined the measured phenotype of forced vital capacity as a better measure of respiratory function. For spherical power, we derived four binary variables to indicate myopia (spherical power<-0.5) and hypertropia (spherical power>0.5) in each eye.

Supplementary Table 10: Effect estimates with 95% confidence intervals examining the causal association between family medical history and Alzheimer's disease using Mendelian Randomization

Outcome	Instruments						Method			
	GWAS	SNPs	F-statistic	Q	p-value for Q statistic	I^2_{gx}	IVW (95% CI)	Egger (95% CI)	p for IVW*	p for Egger intercept
**Maternal history of diabetes**	IEU	24	57.66	27.24	0.25	0.96	1.01 (0.90,1.15)	1.02 (0.76,1.37)	0.94	0.96
**Maternal history of high blood pressure**	IEU	29	40.49	30.20	0.35	0.57	0.92 (0.76,1.11)	1.56 (0.68,3.58)	0.81	0.22
**Paternal history of chronic bronchitis/emphysema**	IEU	4	50.31	4.68	0.20	0.77	0.77 (0.51,1.16)	1.02 (0.25,4.10)	0.56	0.58

All statistical tests were two-sided.

*P represents an adjusted p-value threshold after correction for multiple testing, controlling for a 5% false discovery rate.

Abbreviations: IVW, inverse-variance weighted; GWAS, genome-wide association study

Supplementary Table 11: Effect estimates with 95% confidence intervals examining the causal association between medical history and Alzheimer's disease using Mendelian Randomization

Outcome	Instruments						Method			
	GWAS	SNPs	F-statistic	Q	p for Q statistic	I^2_{gX}	IVW estimate (95% CI)	Egger (95% CI)	p for IVW*	p for Egger intercept
**Aortocoronary bypass graft**	IEU	6	84.61	6.94	0.23	0.91	1.00 (0.93,1.07)	0.91 (0.76, 1.08)	0.99	0.31
**Cholecystectomy**	IEU	39	133.24	60.00	0.01	0.99	1.02 (0.97,1.07)	1.01 (0.95,1.09)	0.81	0.86
**Diagnosis of angina**	IEU	23	53.32	23.73	0.36	0.88	0.96 (0.88,1.04)	0.97 (0.77,1.20)	0.63	0.93
**Diagnosis of atherosclerotic heart disease**	IEU	26	66.34	56.70	0.0003	0.92	0.98 (0.89,1.07)	0.97 (0.78,1.20)	0.89	0.92
**Diagnosis of heart attack**	IEU	13	68.13	16.62	0.16	0.97	0.95 (0.87,1.04)	0.98 (0.76,1.26)	0.63	0.82
**Diagnosis of heart attack/myocardial infarction (self-reported)**	IEU	14	66.45	18.13	0.15	0.97	0.94 (0.86,1.03)	0.99 (0.78,1.27)	0.56	0.65
**Diagnosis of high cholesterol (self-reported)**	IEU	75	94.18	108.01	0.006	0.97	0.95 (0.88,1.02)	1.03 (0.89,1.18)	0.56	0.21
**Diagnosis of pure hypercholesterolaemia**	IEU	15	83.99	9.98	0.76	0.96	0.96 (0.88, 1.06)	0.90 (0.73,1.12)	0.81	0.51
**Use of aspirin**	IEU	12	48.16	21.10	0.03	0.72	1.06 (0.78, 1.43)	0.74 (0.30,1.84)	0.89	0.43
**Use of atenolol**	IEU	12	43.71	20.33	0.04	0.87	1.03 (0.87,1.23)	0.71 (0.32,1.58)	0.89	0.37
**Use of ezetimibe**	IEU	8	54.46	6.80	0.45	0.96	0.99 (0.94,1.05)	0.94 (0.79,1.11)	0.89	0.54
**Use of lipitor**	IEU	5	35.70	4.73	0.32	0	1.01 (0.91,1.11)	0.85 (0.46,1.58)	0.94	0.63

**Use of simvastatin**	IEU	38	64.54	65.27	0.003	0.95	0.98 (0.86, 1.11)	0.93 (0.66, 1.29)	0.89	0.74
**Wheeze/whistling**	IEU	45	55.04	68.98	0.01	0.94	1.03 (0.98,1.08)	1.08 (0.90,1.30)	0.20	0.63

All statistical tests were two-sided. *P represents an adjusted p-value threshold after correction for multiple testing, controlling for a 5% false discovery rate.

Abbreviations: IVW, inverse-variance weighted; GWAS, genome-wide association study

Supplementary Table 12: Effect estimates with 95% confidence intervals examining the causal association between physical measures and **Alzheimer's disease using Mendelian Randomization**

Outcome	Instruments						Method			
	GWAS	SNPs	F-statistic	Q-statistic	p for Q statistic	I^2_{gX}	IVW estimate (95% CI)	Egger (95% CI)	p for IVW *	p for Egger intercept
**Basal metabolic rate**	IEU	524	81.50	645.79	$1.89x10^{-4}$	0.96	0.75 (0.66, 0.85)	0.77 (0.56, 1.05)	0.56	0.85
**Body fat percentage**	IEU	378	58.21	502.64	$1.51x10^{-5}$	0.93	0.84 (0.72, 0.97)	0.70 (0.43, 1.13)	0.30	0.43
**Body mass index**	Yengo et al [31]	492	70.17	568.69	$8.65x10^{-3}$	0.90	0.99 (0.90, 1.10)	0.88 (0.66, 1.17)	0.89	0.38
**Diastolic blood pressure**	Evangelou et al[34]	442	79.16	550.91	$2.77x10^{-4}$	0.95	0.99 (0.98, 1.00)	0.99 (0.96, 1.01)	0.56	0.83
**Forced vital capacity**	IEU	282	62.24	407.47	$1.16x10^{-6}$	0.92	0.78 (0.67,0.91)	0.79 (0.51,1.22)	0.03	0.97
**Hip circumference**	IEU	400	79.16	555.98	$3.13x10^{-7}$	0.95	0.88 (0.79, 0.98)	0.72 (0.53, 0.97)	0.05	0.16
**Hypertropia (left eye)**	IEU	55	57.02	74.86	0.03	0.93	1.02 (0.96, 1.09)	0.93 (0.76, 1.15)	0.89	0.36
**Hypertropia (right eye)**	IEU	61	52.29	85.79	0.02	0.92	1.05 (0.98, 1.12)	0.97 (0.77, 1.21)	0.94	0.44
**Myopia (left eye)**	IEU	76	56.63	99.75	0.03	0.94	0.97 (0.91, 1.03)	1.01 (0.84, 1.20)	0.92	0.64
**Myopia (right eye)**	IEU	80	56.43	112.09	0.009	0.94	0.96 (0.90, 1.01)	0.97 (0.81, 1.16)	0.99	0.91
**Pulse rate (automated reading)**	IEU	284	83.58	366.00	$7.28x10^{-4}$	0.96	1.00 (0.90, 1.11)	1.02 (0.82, 1.28)	0.81	0.80
**Trunk fat percentage**	IEU	368	57.84	489.30	$1.93x10^{-5}$	0.93	0.86 (0.75, 0.97)	0.67 (0.45, 1.01)	0.22	0.21
**Waist circumference**	IEU	356	57.36	511.36	$9.91x10^{-8}$	0.94	0.85 (0.74, 0.98)	0.75 (0.50, 1.13)	0.89	0.53

**Whole body fat mass**	IEU	406	62.36	540.82	$6.91x10^{-6}$	0.94	0.89 (0.80, 0.99)	0.81 (0.59, 1.19)	0.30	0.49
**Whole body fat-free mass**	IEU	543	84.47	665.37	$2.20x10^{-4}$	0.96	0.78 (0.69, 0.88)	0.79 (0.58, 1.07)	0.30	0.94
**Whole body water mass**	IEU	553	82.80	699.22	$1.99x10^{-5}$	0.96	0.81 (0.71, 0.91)	0.74 (0.54, 1.02)	0.89	0.58

All statistical tests were two-sided.

*P represents an adjusted p-value threshold after correction for multiple testing, controlling for a 5% false discovery rate.

Abbreviations: IVW, inverse-variance weighted; GWAS, genome-wide association study

Supplementary Table 13: Effect estimates with 95% confidence intervals examining the causal association between cognitive and brain-related measures and Alzheimer's disease using Mendelian Randomization

Outcome	Instruments						Method			
	GWAS	SNPs	F-statistic	Q-statistic	p for Q-statistic	I^2_{gX}	IVW/Wald ratio (95% CI)	p for IVW/Wald ratio*	Egger (95% CI)	p for Egger intercept
**Duration to complete trail in alphanumeric path test**	IEU	8	38.32	4.93	0.67	0.82	1.34 (0.88,2.04)	0.56	2.98 (0.45,19.78)	0.43
**Duration to entering value in symbol digit substitution test**	IEU	5	36.11	5.13	0.27	0.94	1.53 (0.79,2.97)	0.56	2.75 (0.04,174.60)	0.80
**Fluid intelligence score**	IEU	74	40.19	131.54	$3.22x10^{-5}$	0.83	0.74 (0.59, 0.91)	0.05	0.74 (0.26, 2.10)	0.99
**Interval between previous point and current one in alphanumeric path test**	IEU	8	40.49	4.34	0.63	0	1.34 (0.92, 1.96)	0.89	3.08 (0.41, 23.02)	0.71
**Number of correct matches in round**	IEU	1	30.10	-	-	-	0.31 (0.5, 1.91)	0.56	-	-
**Number of symbol digit matches attempted**	IEU	5	33.74	4.75	0.31	0.86	0.83 (0.44,1.58)	0.89	0.02 (0.0002, 1.71)	0.20
**Number of symbol digit matches made correctly**	IEU	3	32.54	7.06	0.03	0.29	0.60 (0.15,2.33)	0.81	0.15 ($1.15x10^{-6}$,18937.6)	0.85
**Time to complete round in pairs matching test**	IEU	76	39.66	127.30	0.0002	0.89	1.23 (0.87,1.75)	0.61	0.14 (0.02,0.80)	0.02

All statistical tests were two-sided.

*P represents an adjusted p-value threshold after correction for multiple testing, controlling for a 5% false discovery rate.

Abbreviations: IVW, inverse-variance weighted; GWAS, genome-wide association study

Supplementary Table 14: Effect estimates with 95% confidence intervals examining the causal association between biological measures and Alzheimer's disease using Mendelian Randomization

Outcome	Instruments						Method			
	**GWAS**	**SNPs**	**F-statistic**	**Q-statistic**	**p for Q statistic**	I^2_{Gx}	**IVW (95% CI)**	**Egger (95% CI)**	**p for IVW***	**p for Egger intercept**
**Red blood cell count**	IEU	419	108.96	558.69	4.72x10-6	0.97	1.04 (0.94, 1.14)	1.12 (0.91, 1.37)	0.95	0.42
**Red blood cell distribution width**	IEU	353	143.30	462.64	6.58x10-4	0.98	0.96 (0.89, 1.04)	1.08 (0.94, 1.24)	0.61	0.05
**Haematocrit percentage**	IEU	332	93.25	465.67	1.43x10-6	0.97	0.98 (0.87,1.10)	1.00 (0.79,1.27)	0.95	0.85
**Haemoglobin concentration**	IEU	346	94.63	465.69	1.51x10-5	0.97	1.09 (0.97,1.23)	0.94 (0.74,1.19)	0.61	0.15
**Monocyte count**	IEU	476	45.65	565.12	0.003	0.93	0.91 (0.81,1.03)	0.98 (0.68,1.39)	0.61	0.69
**Platelet count**	IEU	518	144.64	718.43	9.72x10-9	0.98	1.02 (0.95, 1.09)	0.95 (0.84, 1.08)	0.58	0.25
**Platelet crit**	IEU	486	122.79	623.28	2.09x10-5	0.98	1.00 (0.93, 1.08)	0.99 (0.86, 1.15)	0.93	0.84
**Reticulocyte volume**	IEU	376	158.85	514.91	2.04x10-6	0.99	1.03 (0.96,1.11)	1.05 (0.92,1.19)	0.93	0.74
**Mean sphered cell volume**	IEU	385	146.35	529.88	1.08x10-6	0.99	1.04 (0.96, 1.12)	1.14 (1.00, 1.31)	0.87	0.09

All statistical tests were two-sided.

[*]P represents an adjusted p-value threshold after correction for multiple testing, controlling for a 5% false discovery rate.

Abbreviations: IVW, inverse-variance weighted; GWAS, genome-wide association study

Supplementary Table 15: Effect estimates with 95% confidence intervals examining the causal association between dietary choices and Alzheimer's disease using Mendelian Randomization

Outcome	Instruments						Method			
	GWAS	SNPs	F-statistic	Q-statistic	p for Q-statistic	I^2_{Gx}	IVW estimate (95% CI)	Egger (95% CI)	p for IVW*	p for Egger intercept
**Cereal intake**	IEU	38	45.59	53.72	0.04	0.88	0.78 (0.52, 1.17)	3.14 (0.55, 18.11)	0.61	0.12
**Dried fruit intake**	IEU	40	41.40	53.66	0.06	0.84	0.76 (0.51, 1.12)	2.18 (0.34, 13.88)	0.56	0.26
**Fresh fruit intake**	IEU	51	46.19	70.29	0.03	0.86	1.08 (0.77, 1.52)	0.89 (0.27, 2.93)	0.89	0.74
**Lamb/mutton intake**	IEU	30	38.46	38.04	0.12	0.85	0.90 (0.54, 1.50)	12.15 (1.14, 129.48)	0.81	0.04
**Non-oily fish intake**	IEU	11	44.80	14.93	0.13	0.86	1.14 (0.51,2.57)	0.06 (0.002,2.23)	0.89	0.14
**Oily fish intake**	IEU	60	44.93	62.46	0.35	0.84	0.84 (0.63, 1.12)	1.33 (0.39,4.51)	0.56	0.45
**Pork intake**	IEU	13	37.28	15.24	0.23	0	1.31 (0.63,2.72)	0.07 (0.0004,10.49)	0.81	0.27
**Salad/raw vegeSupplementary Table intake**	IEU	18	39.23	22.83	0.15	0.88	1.90 (0.99, 3.64)	0.65 (0.02, 23.62)	0.56	0.56
**Salt added to food**	IEU	100	51.01	133.09	0.01	0.90	0.83 (0.66, 1.05)	0.97 (0.43, 2.16)	0.56	0.70
**Saturated fat intake**	IEU	1	31.71	-	-	-	1.12 (0.50, 3.06)	-	0.89	-
**Use of flora**	IEU	6	38.96	4.13	0.53	0.87	0.91 (0.71, 1.16)	0.92 (0.25, 3.36)	0.89	0.98
**Use of spreadable butter**	IEU	15	32.8	15.26	0.36	0.88	0.92 (0.65, 1.30)	0.04 (0.002, 0.68)	0.81	0.05
**Use of white bread**	IEU	27	32.87	45.54	0.01	0.85	1.08 (0.84, 1.38)	1.49 (0.25, 8.88)	0.94	0.72
**Variation in diet**	IEU	15	36.67	16.32	0.29	0.87	0.63 (0.32,1.23)	0.05 (0.0003,9.07)	0.49	0.36

**Water intake**	IEU	40	49.46	36.66	0.87	0.93	0.87 (0.62, 1.21)	0.24 (0.10, 0.62)	0.68	0.01

All statistical tests were two-sided. *P represents an adjusted p-value threshold after correction for multiple testing, controlling for a 5% false discovery rate. Abbreviations: IVW, inverse-variance weighted; GWAS, genome-wide association study

Supplementary Table 16: Effect estimates with 95% confidence intervals examining the causal association between lifestyle factors and Alzheimer's disease using Mendelian Randomization

Outcome	Instruments						Method			
	**GWAS**	**SNPs**	**F-statistic**	**Q-statistic**	**p for Q-statistic**	**I^2_{gx}**	**IVW (95% CI)**	**p for IVW***	**Egger (95% CI)**	**p for Egger intercept**
**Frequency of stair climbing in last 4 weeks**	IEU	17	33.50	21.78	0.15	0	0.82 (0.42, 1.61)	0.82	35.17 (0.18,6700.51)	0.18
**Frequency of walking for pleasure in last 4 weeks**	IEU	8	34.33	11.67	0.11	0	1.47 (0.60,3.59)	0.68	0.003 (1.17×10^{-6},7.07)	0.17
**Getting up in morning**	IEU	74	42.49	110.41	0.003	0.86	1.34 (0.97,1.85)	0.34	1.10(0.35,3.46)	0.72
**Napping during the day**	IEU	93	46.35	99.93	0.27	0.89	0.77 (0.61, 0.97)	0.23	0.31 (0.13,0.74)	0.04
**Number of days/week of MPA**	IEU	15	35.79	15.75	0.33	0.76	2.69 (1.39, 5.17)	0.04	10.57 (0.02, 7062.04)	0.68
**Number of days/week of VPA**	IEU	10	39.00	24.67	0.003	0	1.02 (0.33, 3.18)	0.99	2446.01 (0.02,3.33×10^{8})	0.99
**Sleeplessness / insomnia**	IEU	39	45.09	35.01	0.61	0.84	1.06 (0.75,1.50)	0.84	0.83 (0.23,2.99)	0.70
**Sleep duration**	IEU	68	42.78	119.70	8.10×10^{-5}	0.91	0.97 (0.67,1.39)	0.93	0.55 (0.13,2.23)	0.41
**Usual walking pace**	IEU	56	40.33	68.83	0.10	0.88	0.98 (0.69,1.37)	0.93	0.55 (0.13,2.28)	0.42

All statistical tests were two-sided.*P represents an adjusted p-value threshold after correction for multiple testing, controlling for a 5% false discovery rate. Abbreviations: IVW, inverse-variance weighted; GWAS, genome-wide association study

Supplementary Table 17: Effect estimates with 95% confidence intervals examining the causal association between factors implicated in Alzheimer's disease in previous studies and Alzheimer's disease using Mendelian Randomization

Outcome	Instruments						Method			
	GWAS	SNPs	F-statistic	Q-statistic	p for Q-statistic	I^2_{gx}	IVW/Wald ratio (95% CI)	p for Wald ratio/IWV†	Egger (95% CI)	p for Egger intercept
A levels/AS qualifications	IEU	85	40.36	87.14	0.39	0.86	0.78 (0.70,0.87)	$4.78X10^{-4}$	0.88 (0.47,1.67)	0.71
College degree	IEU	245	46.77	330.44	$1.87x10^{-4}$	0.89	0.82 (0.76,0.88)	$9.21x10^{-6}$	0.76 (0.56,1.04)	0.65
Hearing aid user	IEU	9	34.92	6.75	0.56	0.92	1.03 (0.89,1.19)	0.84	1.21 (0.26,5.60)	0.84
Intake of sugar added to tea	IEU	1	43.29	-	-	-	1.35 (0.43,4.25)	0.84	-	-
O levels qualification	IEU	21	35.59	33.70	0.03	0.36	0.82 (0.58,1.15)	0.53	0.48 (0.03,6.74)	0.69
Other professional qualifications (e.g. teaching)	IEU	18	36.34	19.84	0.28	0.76	0.78 (0.59,1.03)	0.36	1.33 (0.23,7.78)	0.56
Pack years of smoking	IEU	10	77.56	10.79	0.29	0.97	0.86 (0.60,1.22)	0.68	0.34 (0.16,0.70)	0.02
Social activities: pub socials	IEU	18	42.35	29.21	0.03	0.84	1.05 (0.78, 1.43)	0.84	0.75 (0.35, 1.58)	0.34
Social activities: religious groups	IEU	22	37.10	24.51	0.27	0.85	0.84 (0.69,1.01)	0.31	0.61 (0.13,2.90)	0.69
Speech-reception-threshold (SRT) estimate (left)	IEU	1	32.23	-	-	-	0.23 (0.06,0.91)	0.23	-	-
Speech-reception-threshold (SRT) estimate (right)	IEU	1	33.42	-	-	-	0.79 (0.21,2.97)	0.84	-	-

**Systolic blood pressure**	Evangelou et al[34]	437	74.90	632.65	$2.03x10^{-9}$	0.94	1.00 (0.99, 1.00)	0.49	0.99 (0.97, 1.01)	0.36

All statistical tests were two-sided.
*P represents an adjusted p-value threshold after correction for multiple testing, controlling for a 5% false discovery rate.
Abbreviations: IVW, inverse-variance weighted; GWAS, genome-wide association study**.** *Wald ratios are presented only when there is 1 SNP to instrument the exposure

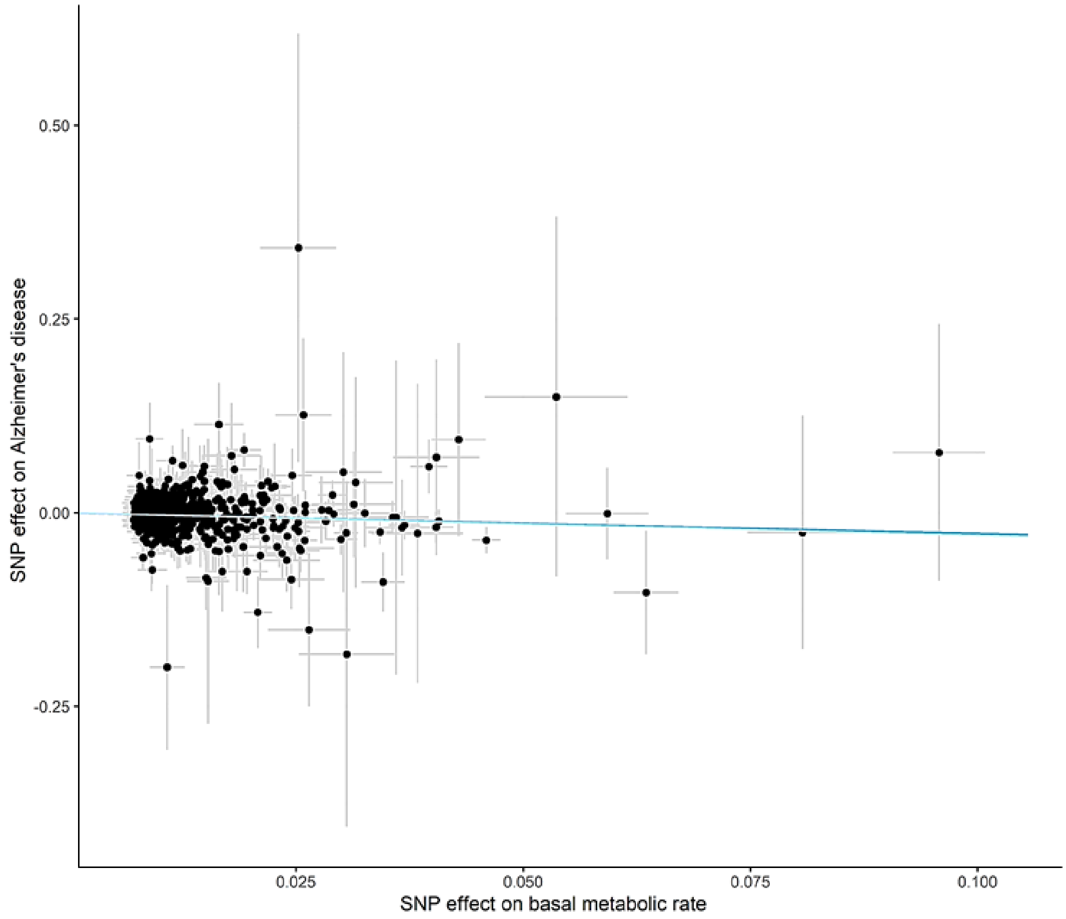

Supplementary Figure 1. Plot displaying the effect of each corresponding SNP on basal metabolic rate (N=454,874) and Alzheimer's disease (N_{cases}=24,087, $N_{controls}$=55,058). Light blue line represents the inverse variance weighted slope and dark blue line represents the Egger slope. Effect estimates are reported per SD increase in the exposure and error bars represent 95% confidence intervals.

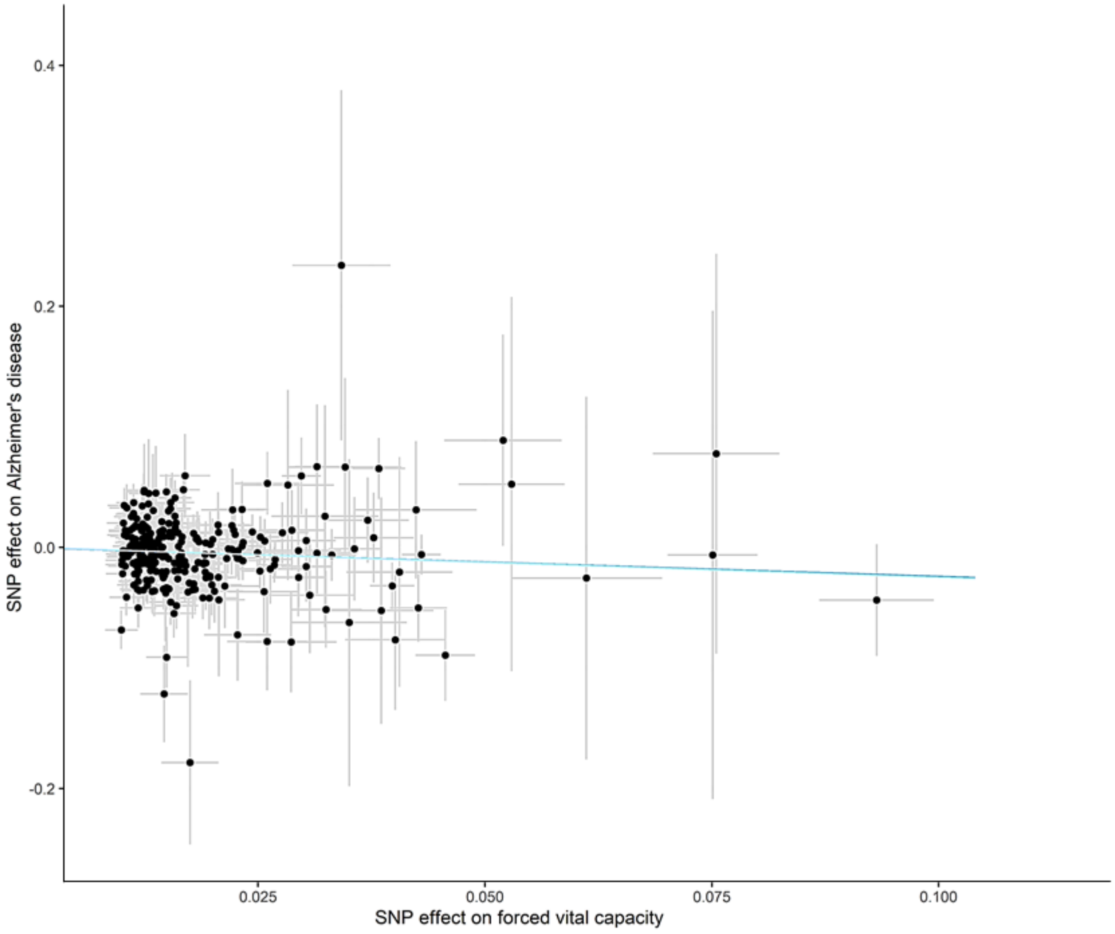

Supplementary Figure 19. Plot displaying the effect of each corresponding SNP on forced vital capacity (N=345,665) and Alzheimer's disease (N_{cases}=24,087, $N_{controls}$=55,058). Light blue line represents the inverse variance weighted slope and dark blue line represents the Egger slope. Effect estimates are reported per SD increase in the exposure and error bars represent 95% confidence intervals.

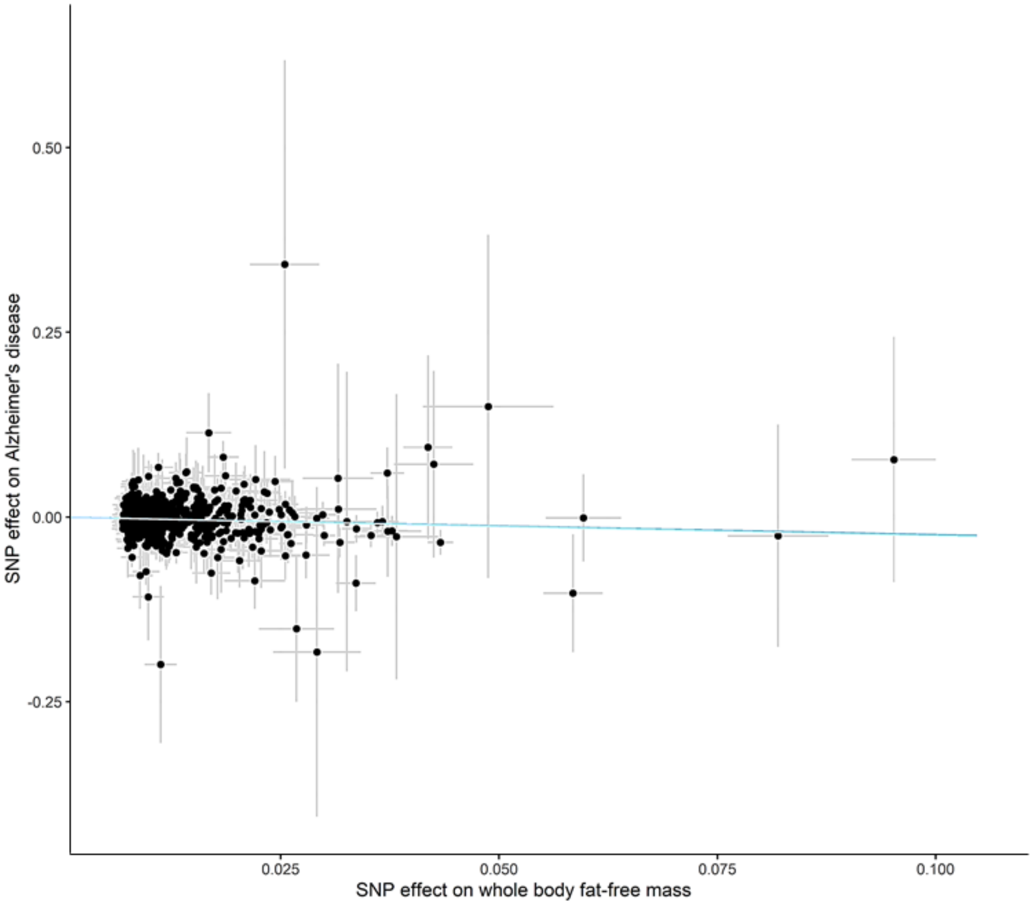

Supplementary Figure 20. Plot displaying the effect of each corresponding SNP on whole body fat-free mass (N=454,850) and Alzheimer's disease (N_{cases}=24,087, $N_{controls}$=55,058). Light blue line represents the inverse variance weighted slope and dark blue line represents the Egger slope. Effect estimates are reported per SD increase in the exposure and error bars represent 95% confidence intervals.

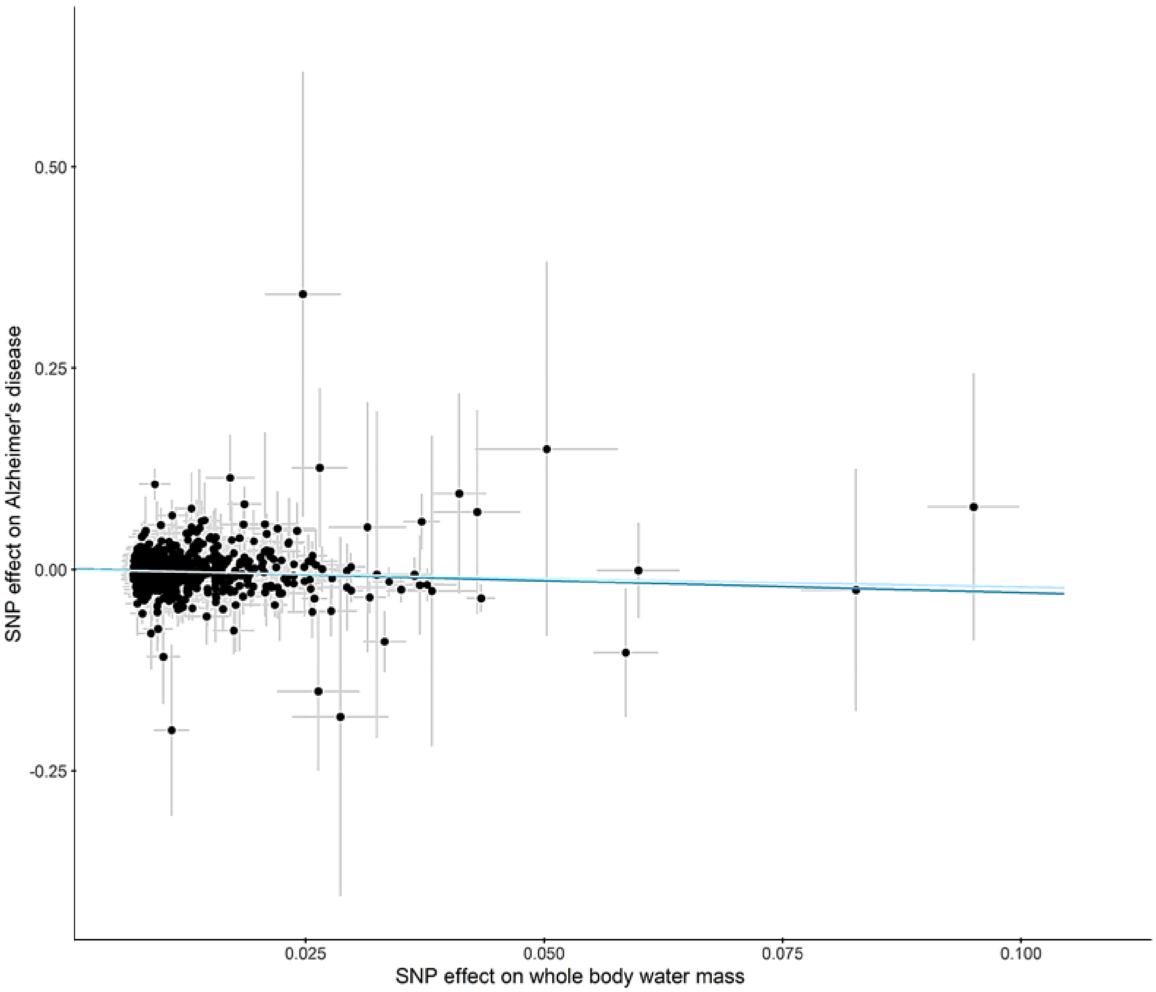

Supplementary Figure 21. Plot displaying the effect of each corresponding SNP on whole body water mass (N=454,888) and Alzheimer's disease (N_{cases}=24,087, $N_{controls}$=55,058). Light blue line represents the inverse variance weighted slope and dark blue line represents the Egger slope. Effect estimates are reported per SD increase in the exposure and error bars represent 95% confidence intervals.

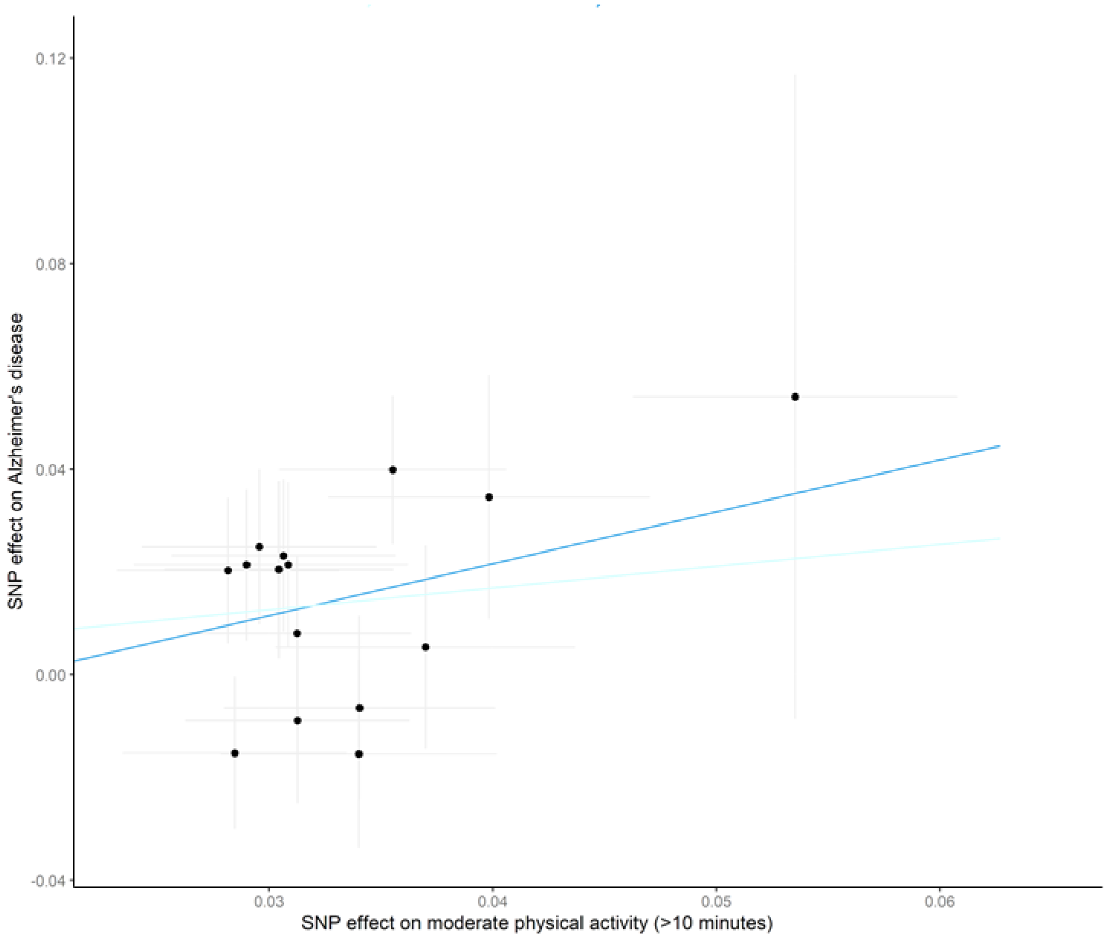

Supplementary Figure 22. Plot displaying the effect of each corresponding SNP on moderate physical activity (>10 minutes) (N=440,266) and Alzheimer's disease (N_{cases}=24,087, $N_{controls}$=55,058). Light blue line represents the inverse variance weighted slope and dark blue line represents the Egger slope. Effect estimates are reported per SD increase in category of the exposure and error bars represent 95% confidence intervals.

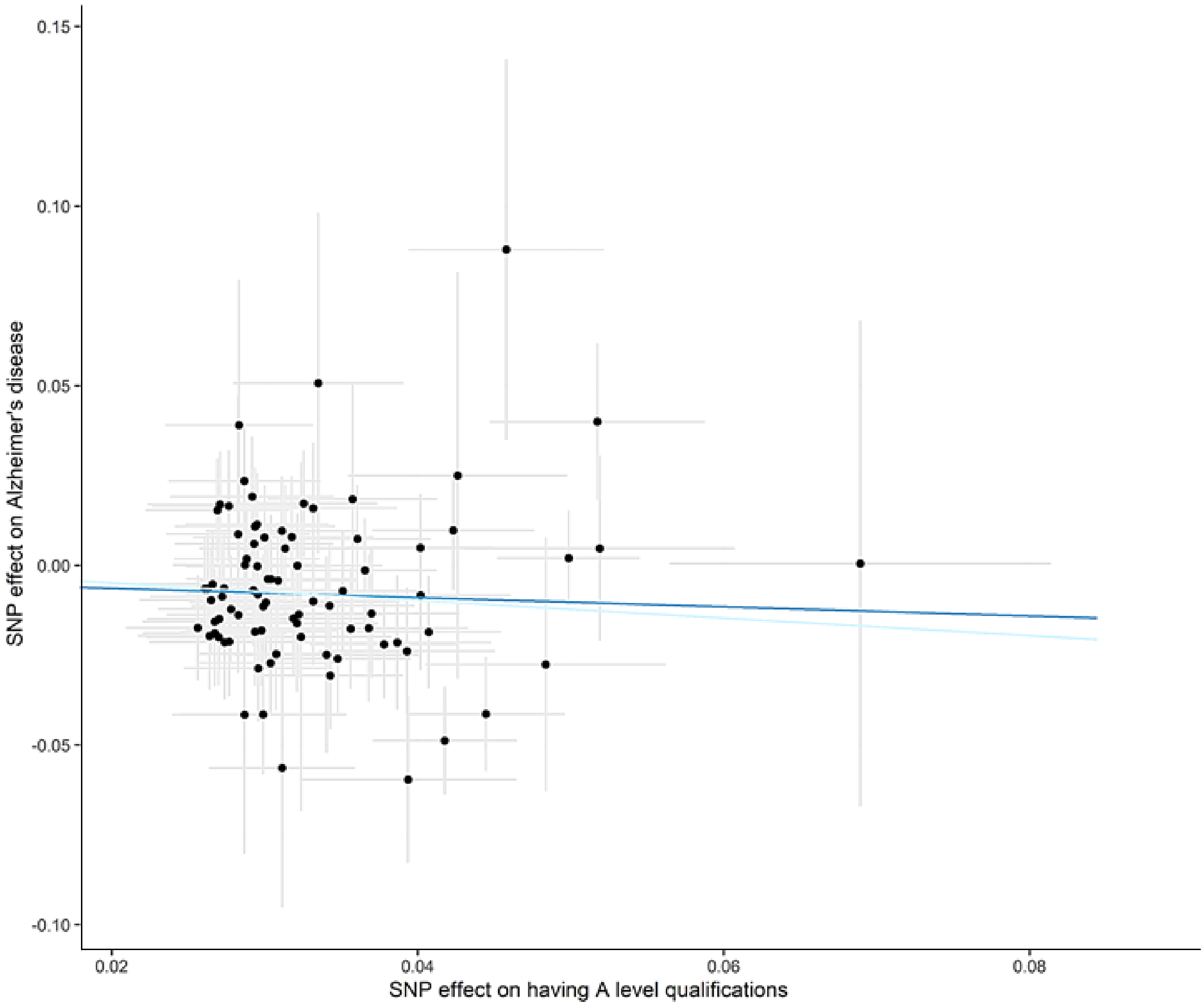

Supplementary Figure 23. Plot displaying the effect of each corresponding SNP on A-level qualifications (N=458,079) and Alzheimer's disease (N_{cases}=24,087, $N_{controls}$=55,058). Light blue line represents the inverse variance weighted slope and dark blue line represents the Egger slope. Effect estimates are reported per SD increase in the exposure and error bars represent 95% confidence intervals.

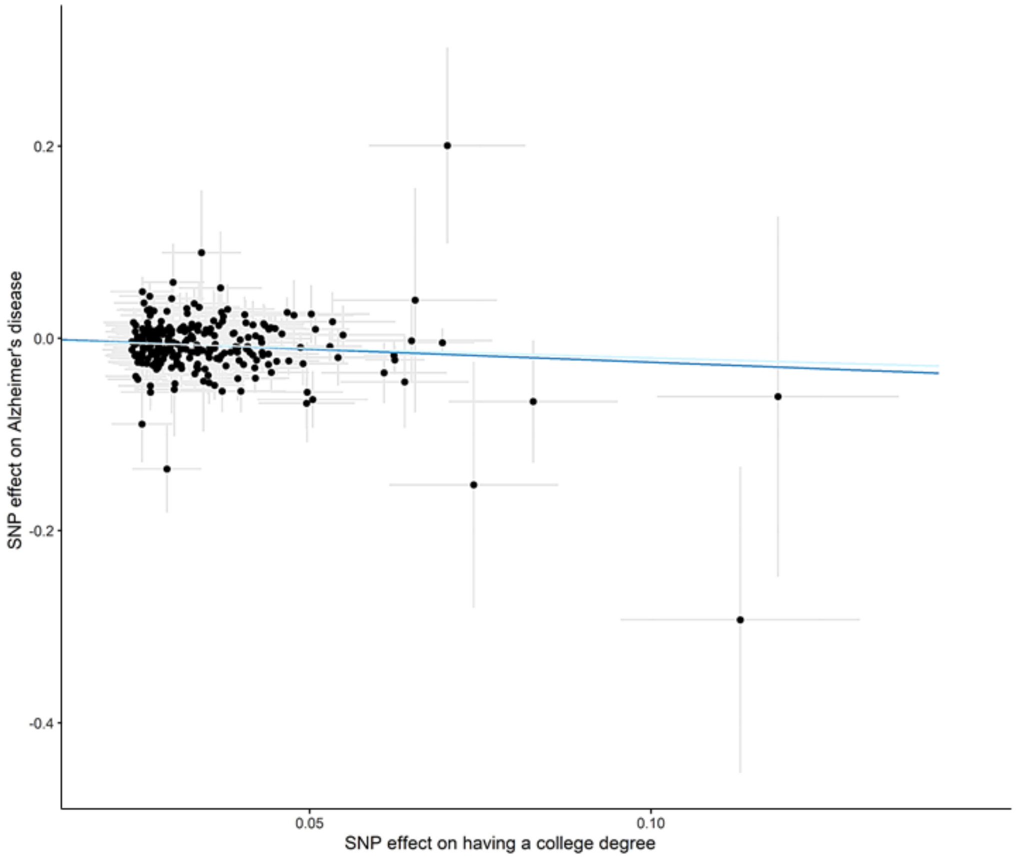

Supplementary Figure 24. Plot displaying the effect of each corresponding SNP on having a college degree (N=458,079) and Alzheimer's disease (N_{cases}=24,087, $N_{controls}$=55,058). Light blue line represents the inverse variance weighted slope and dark blue line represents the Egger slope. Effect estimates are reported per SD increase in the exposure and error bars represent 95% confidence intervals.

Supplementary references

1. Bycroft C, Freeman C, Petkova D, et al. Genome-wide genetic data on ~500,000 UK Biobank participants. *doi.org*. Published online 2017:166298. doi:10.1101/166298

2. O'Connell J, Sharp K, Shrine N, et al. Haplotype estimation for biobank-scale data sets. *Nature Genetics*. 2016;48(7):817-820. doi:10.1038/ng.3583

3. Huang J, Howie B, McCarthy S, et al. Improved imputation of low-frequency and rare variants using the UK10K haplotype reference panel. *Nature Communications 2015 6:1*. 2015;6(1):1-9. doi:10.1038/ncomms9111

4. Howie BN, Donnelly P, Marchini J. A flexible and accurate genotype imputation method for the next generation of genome-wide association studies. *PLoS Genetics*. 2009;5(6). doi:10.1371/journal.pgen.1000529

5. Mitchell R, Hemani G, Dudding T, Paternoster L. UK Biobank Genetic Data: MRC-IEU Quality Control, Version 1. doi:doi.org doi:10.5523/bris.3074krb6t2frj29yh2b03x3wxj

6. Lambert JC, Ibrahim-Verbaas CA, Harold D, et al. Meta-analysis of 74,046 individuals identifies 11 new susceptibility loci for Alzheimer's disease. *Nature Genetics*. 2013;45(12):1452-1458.

7. Harold D, Abraham R, Hollingworth P, et al. Genome-wide association study identifies variants at CLU and PICALM associated with Alzheimer's disease. *Nature Genetics*. 2009;41(10):1088-1093. doi:10.1038/ng.440

8. Hollingworth P, Harold D, Sims R, et al. Common variants at ABCA7, MS4A6A/MS4A4E, EPHA1, CD33 and CD2AP are associated with Alzheimer's disease. *Nat Genet*. 2011;43(5):429-435. doi:10.1038/ng.803

9. Seshadri S, Beiser A, Selhub J, et al. Plasma homocysteine as a risk factor for dementia and Alzheimer's disease. *N Engl J Med*. 2002;346(7):476-483. doi:10.1056/NEJMoa011613

10. Naj AC, Jun G, Beecham GW, et al. Common variants at MS4A4/MS4A6E, CD2AP, CD33 and EPHA1 are associated with late-onset Alzheimer's disease. *Nat Genet*. 2011;43(5):436-441.

11. Li Y, Willer CJ, Ding J, Scheet P, Abecasis GR. MaCH: Using sequence and genotype data to estimate haplotypes and unobserved genotypes. *Genetic Epidemiology*. 2010;34(8):816-834. doi:10.1002/gepi.20533

12. Jansen I, Savage J, Watanabe K, et al. Genetic meta-analysis identifies 9 novel loci and functional pathways for Alzheimers disease risk. *bioRxiv*. Published online January 1, 2018:258533. doi:10.1101/258533

13. Allen NE, Sudlow C, Peakman T, Collins R. UK Biobank Data: Come and Get It. *Science Translational Medicine*. 2014;6(224):224ed4-224ed4. doi:10.1126/scitranslmed.3008601

14. Collins R. What makes UK Biobank special? *The Lancet*. 2012;379(9822):1173-1174. doi:10.1016/S0140-6736(12)60404-8

15. Beecham GW, Bis JC, Martin ER, et al. Clinical/Scientific Notes: The Alzheimer's disease sequencing project: Study design and sample selection. *Neurology: Genetics*. 2017;3(5). doi:10.1212/NXG.0000000000000194

16. Jansen IE, Savage JE, Watanabe K, et al. Genome-wide meta-analysis identifies new loci and functional pathways influencing Alzheimer's disease risk. *Nature Genetics*. 2019;51(3):404-413. doi:10.1038/s41588-018-0311-9

17. Purcell S, Neale B, Todd-Brown K, et al. PLINK: A Tool Set for Whole-Genome Association and Population-Based Linkage Analyses. *American Journal of Human Genetics*. 2007;81(3):559. doi:10.1086/519795

18. Millard LAC, Davies NM, Gaunt TR, Davey Smith G, Tilling K. Software Application Profile: PHESANT: a tool for performing automated phenome scans in UK Biobank. *International Journal of Epidemiology*. 2018;47(1):29-35. doi:10.1093/ije/dyx204

19. Cai Q, Xin Z, Zuo L, Li F, Liu B. Alzheimer's disease and rheumatoid arthritis: A mendelian randomization study. *Frontiers in Neuroscience*. 2018;12(SEP). doi:10.3389/fnins.2018.00627

20. Judge A, Garriga C, Arden NK, et al. Protective effect of antirheumatic drugs on dementia in rheumatoid arthritis patients. *Alzheimer's and Dementia: Translational Research and Clinical Interventions*. 2017;3(4):612-621. doi:10.1016/j.trci.2017.10.002

21. Chou RC, Kane M, Ghimire S, Gautam S, Gui J. Treatment for Rheumatoid Arthritis and Risk of Alzheimer's Disease: A Nested Case-Control Analysis. *CNS Drugs*. 2016;30(11):1111-1120. doi:10.1007/s40263-016-0374-z

22. Åsvold BO, Langhammer A, Rehn TA, et al. Cohort Profile Update: The HUNT Study, Norway. *medRxiv*. Published online October 19, 2021:2021.10.12.21264858. doi:10.1101/2021.10.12.21264858

23. Holmen J, Midthjell K, Krüger Ø, et al. The Nord-Trøndelag Health Study 1995-97 (HUNT 2): Objectives, contents, methods and participation. *Norsk Epidemiologi*. 2003;13(1):19-32.

24. Krokstad S, Langhammer A, Hveem K, et al. Cohort Profile: the HUNT Study, Norway. *Int J Epidemiol*. 2013;42(4):968-977. doi:10.1093/IJE/DYS095

25. Ferreira MA, Vonk JM, Baurecht H, et al. Shared genetic origin of asthma, hay fever and eczema elucidates allergic disease biology. *Nat Genet*. 2017;49(12):1752-1757. doi:10.1038/NG.3985

26. Brumpton BM, Graham S, Surakka I, et al. The HUNT Study: a population-based cohort for genetic research. *medRxiv*. Published online December 25, 2021:2021.12.23.21268305. doi:10.1101/2021.12.23.21268305

27. Wang C, Zhan X, Bragg-Gresham J, et al. Ancestry estimation and control of population stratification for sequence-based association studies. *Nat Genet*. 2014;46(4):409-415. doi:10.1038/NG.2924

28. Li JZ, Absher DM, Tang H, et al. Worldwide human relationships inferred from genome-wide patterns of variation. *Science*. 2008;319(5866):1100-1104. doi:10.1126/SCIENCE.1153717

29. Yang J, Lee SH, Goddard ME, Visscher PM. GCTA: A Tool for Genome-wide Complex Trait Analysis. *American Journal of Human Genetics*. 2011;88(1):76. doi:10.1016/J.AJHG.2010.11.011

30. Loh PR, Tucker G, Bulik-Sullivan BK, et al. Efficient Bayesian mixed-model analysis increases association power in large cohorts. *Nature Genetics*. 2015;47(3):284-290. doi:10.1038/ng.3190

31. Yengo L, Sidorenko J, Kemper KE, et al. Meta-analysis of genome-wide association studies for height and body mass index in ∼700000 individuals of European ancestry. *Hum Mol Genet*. 2018;27(20):3641-3649. doi:10.1093/hmg/ddy271

32. Shungin D, Winkler T, Croteau-Chonka DC, et al. New genetic loci link adipose and insulin biology to body fat distribution. *Nature*. Published online 2015. doi:10.1038/nature14132

33. Loh PR, Kichaev G, Gazal S, Schoech AP, Price AL. Mixed-model association for biobank-scale datasets. *Nature Genetics*. 2018;50(7):906-908. doi:10.1038/s41588-018-0144-6

34. Evangelou E, Warren HR, Mosen-Ansorena D, et al. Genetic analysis of over 1 million people identifies 535 new loci associated with blood pressure traits. *Nature Genetics 2018 50:10*. 2018;50(10):1412-1425. doi:10.1038/s41588-018-0205-x
