# Supplementary material for: The causes and consequences of Alzheimer’s disease: phenome-wide evidence from Mendelian randomization
Source: Nat Commun. 2022 Aug 11;13:4726. doi: 10.1038/s41467-022-32183-6 (PMC9372151; doi:10.1038/s41467-022-32183-6)
Supplement: Supplementary file 3 — Description of Additional Supplementary Files [file 41467_2022_32183_MOESM3_ESM.pdf]

Description of Additional Supplementary Files

File Name: Supplementary Data 1
Description: PheWAS of polygenic risk score for tertile 1 participants (ages 39-53 years)

File Name: Supplementary Data 2
Description: PheWAS of polygenic risk score for tertile 2 participants (ages 53-62 years)

File Name: Supplementary Data 3
Description: PheWAS of polygenic risk score for tertile 3 participants (ages 62-72 years)

File Name: Supplementary Data 4
Description: Replication of PheWAS hits in the HUNT study

File Name: Supplementary Data 5
Description: PheWAS of polygenic risk score including the APOE region for the entire UK Biobank sample (N=334,968 participants)

File Name: Supplementary Data 6
Description: PheWAS of polygenic risk score excluding the *APOE* region for the entire UK Biobank sample (N=334,968 participants)

File Name: Supplementary Data 7
Description: Steiger filtering for basal metabolic rate

File Name: Supplementary Data 8
Description: Steiger filtering for whole fat-free mass

File Name: Supplementary Data 9
Description: Steiger filtering for whole body water mass

File Name: Supplementary Data 10
Description: Steiger filtering for forced vital capacity

File Name: Supplementary Data 11
Description: Steiger filtering for self-reported moderate physical activity

File Name: Supplementary Data 12
Description: Steiger filtering for A level qualifications

File Name: Supplementary Data 13
Description: Steiger filtering for college degree qualifications
