# Supplementary material for: The causes and consequences of Alzheimer’s disease: phenome-wide evidence from Mendelian randomization
Source: Nat Commun. 2022 Aug 11;13:4726. doi: 10.1038/s41467-022-32183-6 (PMC9372151; doi:10.1038/s41467-022-32183-6)
Supplement: Supplementary file 5 — Reporting Summary [file 41467_2022_32183_MOESM5_ESM.pdf]

nature portfolio

Corresponding author(s): Roxanna Korologou-Linden

Last updated by author(s): 15-06-2022

Reporting Summary

Nature Portfolio wishes to improve the reproducibility of the work that we publish. This form provides structure for consistency and transparency in reporting. For further information on Nature Portfolio policies, see our Editorial Policies and the Editorial Policy Checklist.

Statistics

For all statistical analyses, confirm that the following items are present in the figure legend, table legend, main text, or Methods section.

n/a	Confirmed	
☐	☒	The exact sample size (*n*) for each experimental group/condition, given as a discrete number and unit of measurement
☐	☒	A statement on whether measurements were taken from distinct samples or whether the same sample was measured repeatedly
☐	☒	The statistical test(s) used AND whether they are one- or two-sided<br>*Only common tests should be described solely by name; describe more complex techniques in the Methods section.*
☐	☒	A description of all covariates tested
☐	☒	A description of any assumptions or corrections, such as tests of normality and adjustment for multiple comparisons
☐	☒	A full description of the statistical parameters including central tendency (e.g. means) or other basic estimates (e.g. regression coefficient) AND variation (e.g. standard deviation) or associated estimates of uncertainty (e.g. confidence intervals)
☐	☒	For null hypothesis testing, the test statistic (e.g. *F*, *t*, *r*) with confidence intervals, effect sizes, degrees of freedom and *P* value noted<br>*Give P values as exact values whenever suitable.*
☒	☐	For Bayesian analysis, information on the choice of priors and Markov chain Monte Carlo settings
☒	☐	For hierarchical and complex designs, identification of the appropriate level for tests and full reporting of outcomes
☐	☒	Estimates of effect sizes (e.g. Cohen's *d*, Pearson's *r*), indicating how they were calculated

Our web collection on statistics for biologists contains articles on many of the points above.

Software and code

Policy information about availability of computer code

Data collection	Data was already available, no software was used for data collection
Data analysis	PHESANT (version 14)<br>R 4.0.3<br>PLINK 1.9<br>GCTA 1.93.3beta2<br>Two sample MR v0.5.6

For manuscripts utilizing custom algorithms or software that are central to the research but not yet described in published literature, software must be made available to editors and reviewers. We strongly encourage code deposition in a community repository (e.g. GitHub). See the Nature Portfolio guidelines for submitting code & software for further information.

Data

Policy information about availability of data

All manuscripts must include a data availability statement. This statement should provide the following information, where applicable:

- Accession codes, unique identifiers, or web links for publicly available datasets
- A description of any restrictions on data availability
- For clinical datasets or third party data, please ensure that the statement adheres to our policy

The data in the current study were provided by the UK Biobank Study (www.ukbiobank.ac.uk), received under the data request application no. 16729. The Alzheimer's disease GWAS included summary statistics from IGAP, ADSP, and PGC. Summary statistics from IGAP are publicly available at http://web.pasteur-lille.fr/en/recherche/u744/igap/igap_download.php. Summary statistics for ADSP can be obtained through a data access request https://dss.niagads.org/documentation/

data-application-and-submission/application-instructions/. Summary statistics from the PGC consortium are available at https://www.med.unc.edu/pgc/download-results/. The exposure GWAS in the follow-up Mendelian randomization studies were performed by Ben Elsworth and are publicly available at https://gwas.mrcieu.ac.uk. The GWAS on the blood-based biomarkers were performed by Roxanna Korologou-Linden, using the UK Biobank pipeline and can be provided upon request.

Field-specific reporting

Please select the one below that is the best fit for your research. If you are not sure, read the appropriate sections before making your selection.

☒ Life sciences ☐ Behavioural & social sciences ☐ Ecological, evolutionary & environmental sciences

For a reference copy of the document with all sections, see nature.com/documents/nr-reporting-summary-flat.pdf

Life sciences study design

All studies must disclose on these points even when the disclosure is negative.

Sample size

The full UK Biobank sample was divided into three age-stratified subsamples (n=111,656 in each tertile), with the aim to examine the age-varying effects of the polygenic risk score for Alzheimer's disease. We performed PheWAS within each tertile. For the follow-up Mendelian randomization analyses, we include the reference for the exposure GWAS in the Supplementary Information - we used the largest GWAS to date.

Data exclusions

In the PheWAS, UK Biobank participants were excluded due to familial relatedness and non-Caucasian ancestry. In the Mendelian randomization studies, exposure and outcome samples were based on participants of European ancestries. In the Supplementary Information, we provide a reference to the GWAS used for further details.

Replication

The polygenic risk score used genetic variants from a meta-analysis of ADSP, PGC, and IGAP (Phase 1 of the Alzheimer's disease GWAS by Jansen et al (2019). We include the list of SNPs and associated weights in the Supplementary Information. Furthermore we used the HUNT study to replicate the top findings identified in the oldest age group (62-72 years) of our phenome-wide association study of ~125,000 participants, which invited the entire adult (≥20 years) population of Trøndelag. For the replication of the Alzheimer's disease polygenic risk score-outcome associations in the HUNT study, we followed up 33 outcomes (i.e. those variables available in HUNT with sufficient sample numbers for replication) that were found to be associated with the Alzheimer's disease polygenic risk score in UK Biobank. Details can be found in the Results of the manuscript as well as Supplementary Information. Replication was conducted once on the replication sample. The blood-based biomarkers and some lifestyle factors in HUNT closely mirrored the effects in UK Biobank.

Randomization

Polygenic risk scores and Mendelian randomization are methods that use alleles which are randomly allocated at conception as instrumental variables to estimate the causal effect of an exposure on an outcome and are hence less prone to bias of confounding and reverse causation associated with observational studies.

Blinding

Both methods rely on the randomization of the genotype during meiosis, which is analogous to blinding to allocation.

Reporting for specific materials, systems and methods

We require information from authors about some types of materials, experimental systems and methods used in many studies. Here, indicate whether each material, system or method listed is relevant to your study. If you are not sure if a list item applies to your research, read the appropriate section before selecting a response.

Materials & experimental systems

n/a	Involved in the study	
☒	☐	Antibodies
☒	☐	Eukaryotic cell lines
☒	☐	Palaeontology and archaeology
☒	☐	Animals and other organisms
☐	☒	Human research participants
☒	☐	Clinical data
☒	☐	Dual use research of concern

Methods

n/a	Involved in the study	
☒	☐	ChIP-seq
☒	☐	Flow cytometry
☒	☐	MRI-based neuroimaging

Human research participants

Policy information about studies involving human research participants

Population characteristics

In our study, the UK Biobank sample was stratified into three age-ordered sub-samples. The sample is 55% female (39 to 53 years, mean=47.2 years, SD=3.8 years) in tertile 1, 55% female (53 to 62 years, mean=58.03 years, SD=2.4 years) in tertile 2 and 49% female (62 to 72 years, mean-65.3 years, SD=2.7 years) in tertile 3.

Recruitment

The UK Biobank cohort is a population-based cohort of 500,000 participants recruited in the United Kingdom (UK) between

Recruitment	2006 and 2010. Approximately 9.2 million individuals aged 40-69 years who lived within 25 miles (40 km) of one of 22 assessment centres in England, Wales, and Scotland were invited to enter the cohort, and 5.5% participated in the baseline assessment. The representativeness of the UK Biobank cohort was investigated by comparing demographic characteristics of the between non-responders and responders. Sociodemographic, physical, lifestly and health-related traits of the cohort were compared with nationally representative data sources. UK Biobank participants were more likely to be older, to be female, and to live in less socioeconomically deprived areas than non-participants. Compared with the general population, participants were less likely to be obese, to smoke, and to drink alcohol on a daily basis and had fewer self-reported health conditions. We have discussed the limitations and the potential selection bias in the Discussion of the manuscript. Selection bias may occur if those with a lower genetic liability to Alzheimer's disease and a specific trait (e.g. higher education or higher levels of physical activity) are more likely to participate in the study. Such selection could induce an association between genetic liability for Alzheimer's disease and the traits in our study. Additionally, if both the PRS for Alzheimer's disease and the examined traits associate with survival, sampling only living people can induce spurious associations that do not exist in the general population. We advise readers to interpret variables associated with selection or survival with caution, in light of these limitations.
Ethics oversight	The UK Biobank study resource has ethical approval and its own ethics committee (http://wwww.ukbiobank.ac.uk/learn-more-about-uk-biobank/governance/ethics-advisory-committee).

Note that full information on the approval of the study protocol must also be provided in the manuscript.
